# Delta and Omicron: protective measures and SARS-CoV-2 infections in day care centres in Germany in the 4th and 5th wave of the pandemic 2021/2022

**DOI:** 10.1186/s12889-022-14521-x

**Published:** 2022-11-17

**Authors:** Franz Neuberger, Mariana Grgic, Udo Buchholz, Hanna Lena Maly-Motta, Sina Fackler, Ann-Sophie Lehfeld, Walter Haas, Bernhard Kalicki, Susanne Kuger

**Affiliations:** 1 German Youth Institute (DJI), Nockherstr. 2, 81541 Munich, Germany; 2 Robert Koch Institute (RKI), Nordufer 20, 13353 Berlin, Germany

**Keywords:** SARS-CoV-2, COVID-19, Early childhood education and care (ECEC), Germany

## Abstract

**Background:**

During the five waves of the SARS-CoV-2 pandemic so far, German early childhood education and care (ECEC) centres implemented various protective measures, such as wearing a face mask, fixed children-staff groups or regular ventilation. In addition, parents and ECEC staff were increasingly vaccinated throughout 2021. During the 4th wave, variant of concern (VOC) Delta-driven transmission indicators reached record values at the end of 2021. Those values were even exceeded in the 5th wave at the beginning of 2022 when Omicron dominated. We examine which factors facilitated or prevented infection with SARS-CoV-2 in ECEC centres, and if these differed between different phases within wave 4 (Delta) and 5 (Omicron).

**Methods:**

Since August 2020, a weekly online survey among approximately 8000 ECEC managers has been conducted, monitoring both incident SARS-CoV-2 infections and protective measures taken. We included data from calendar week 26/2021 to 05/2022. We estimate the probability of any infections and the number of SARS-CoV-2 infections in children, parents and staff using random-effect-within-between (REWB) panel models for binomial and count data.

**Results:**

While children, parents and staff of ECEC centres with a high proportion of children from families with low socioeconomic status (SES) have a higher risk of infections in the beginning of wave 4 (OR up to 1.99 [1.56; 2.56]), this effect diminishes for children and parents with rising incidences. Protective measures, such as wearing face masks, tend to have more extensive effects with rising incidences in wave 5 (IRR up to 0.87 [0.8; 0.93]). Further, the protective effect of vaccination against infection among staff is decreasing from wave 4 to wave 5 (OR 0.3 [0.16; 0.55] to OR 0.95, [0.84; 1.07, n.s.]). The degree of transmission from staff to child and from staff to parent is decreasing from wave 4 to wave 5, while transmission from child to staff seems to increase.

**Conclusion:**

While Omicron seems to affect children and parents from ECEC centres with families with all SES levels more equally than Delta, the protective effect of vaccination against infection is decreasing and the effect of protective measures like face masks becomes increasingly important. In order to prevent massive closures of ECEC centres due to infection of staff, protective measures should be strictly adhered to, especially to protect staff in centres with a high proportion of children from families with low socioeconomic status.

**Supplementary Information:**

The online version contains supplementary material available at 10.1186/s12889-022-14521-x.

## Background

Germany has been hit by five waves of the SARS-CoV-2 pandemic so far. The first wave started in spring 2020, the second started in calendar week CW40/2020 and lasted until the beginning of 2021 and was immediately followed by the third in spring 2021. While the first three waves, dominated by wild-type (first and second wave) and Alpha variant (third wave) were still relatively moderate, the Delta variant causing the 4th wave at the end of 2021 led to heretofore unparalleled spread and transmission [1]. The Delta wave in Germany was split in two parts, a lighter one after the end of the summer school vacation and a heavy one in November/December 2021, in this paper called waves 4a and 4b. The dramatic increase documented during the Delta wave was even surpassed by the spread of the Omicron variant at the start of 2022.

In the course of the SARS-CoV-2 pandemic, German early childhood education and care (ECEC) centres implemented various protective measures, such as wearing a face mask, fixed children-staff groups or regular ventilation, the regular performance of Covid-19 rapid tests and a prioritised vaccination of staff. Vaccination in Germany began shortly before the beginning of the third wave. Until the beginning of the fifth wave it was not possible to vaccinate 0-5 year old children against COVID-19. While children in that age group were relatively spared in the first and second wave, their incidence rose in the third and fourth wave [1].

In 2020, the German Youth Institute (“Deutsches Jugendinstitut”; DJI) and the Robert Koch Institute (the national public health institute; RKI) joined forces to monitor the situation of preschool children during the pandemic. A nationwide register drawing weekly information directly from approximately 8000 ECEC centres (“ECEC centre registry”) was established by the DJI. Aim of the ECEC centre registry was to monitor incident infections with SARS-CoV-2 and the application of protective measures in ECEC centres during the pandemic. Using data collected from August 2020 until the beginning of June 2021 (CW36/2020-CW22/2021), [2] showed that ECEC centres with more children from families with lower socio-economic status (SES) faced increased infections and that strict adherence to fixed staff-children groups decreased infections. Both effects were stronger during wave 3 (Alpha-dominated) compared to wild-type dominated wave 2.

### Research question

Using unique data from the ECEC centre registry, we investigate the occurrence of SARS-CoV-2 infections among children, parents and staff in ECEC centres in the 4th and 5th wave with reference to social determinants (i.e. the socio-economic status of families of children attending ECEC centres), recommended structural and SARS-CoV-2-protective measures as well as vaccination of pedagogical staff. We analyse which factors contributed or prevented infection with SARS-CoV-2 in ECEC centres, and if these differed between different phases within wave 4 or wave 5, respectively, where on the one hand different conditions (summer and fall vs. winter) and on the other hand different Covid variants (Delta vs. Omicron) prevailed. In addition, possible chains of infection within the centre are taken into account.

### Protective measures and the transmission of SARS-CoV-2 in ECEC centres

In the 4th and 5th wave of the pandemic, ECEC centres in Germany remained open all the time for all groups of children. After three phases of ECEC closures with limited service (for certain groups of children) in the first, second and third wave of the pandemic, further closures were waived due to suspected negative long-term consequences for child development, as studies [3] have shown that ECEC attendance during the pandemic boost growth of cognitive executive functions in 8 to 36 month old children. To ensure the opening, political decisions held on to protective measures in ECEC centres, like group separation, wearing face masks by staff and parents, regular testing and vaccination of staff [2]. In the 4th and 5th wave, regular testing of children on Covid-19 has been introduced, too. In autumn 2021, political initiatives started to promote so-called booster vaccinations (3rd shot) for adults in Germany, which also affected pedagogical staff in ECEC centres.

Overall, transmission of COVID-19 infections in ECEC centres showed a low proportion of young children in their samples with SARS-CoV-2 infection according to serological test results, hence, intrafamily transmission seemed more plausible than transmission within ECEC centres [4]. The German COALA study confirmed a higher secondary attack rate (SAR) in households (53.3%) than in ECEC centres (15.9% with evidence of the Alpha variant and 5.1% with evidence of wild type). Adults had a higher probability of becoming infected than children [5]. For schools, studies reported a low transmission in schools, even during a surge of community infections [6]. Despite this lower likelihood of infection by children, ECEC centres remain epidemiologically and socially relevant as a place where large numbers of adults and children congregate daily. Several studies pointed out health disparities concerning SES of persons [7] and certain ethnic groups [8]. Furthermore a strong relation between the number of infections in children and staff of ECEC centres and SES composition of the attending children was found before in German ECEC centres [2].

Regarding the effects of protective measures, a high COVID-19 policy adherence in general relates to low transmission in community hubs [9]. Certain protective measures in ECEC centres could been shown to prevent infections, such as implementatio of group separation and fixed staff reduces infections in children and staff during the second and third wave of the pandemic in Germany [2]. However, the effects of new protective measures such as the wearing of face masks as well a the effect of vaccination/booster vaccination of staff or regular testing could not yet be tested in this study, as these were not yet included in the version of the questionnaire used at that time. While the overall protective effect of facemasks is without question [10], there are good reasons to believe that the obligation to wear face masks also has protective effects in educational settings. A recent study [11], combining data from ECEC and school settings, found that mask obligations with staff/teachers have effects on the size of outbreaks, i.e. on the number of secondary cases. However, studies focusing on ECEC settings only are rather rare and found no protective effects [12] for staff wearing face masks. However, both studies showed significant protective effects if children were also required to wear masks. This question does not arise in the context of ECEC centres in Germany and was not asked in our study, as, contrary to the US [13], no recommendations were made on mask wearing in the 0-6 age group and children were never obligated to wear masks [11] in Germany.

## Methods

### Study Design

Managers of all 55000 ECEC centres in Germany were contacted by letter and eligible to participate. During 2020 and 2021 a weekly mean of 5200 (9.5%) ECEC centres contributed data. Starting in CW36/2020, managers of all ECEC centres in Germany were asked to fill out a weekly questionnaire. The survey comprises a baseline questionnaire handed out to the ECEC centres at the time point of registration. The baseline questionnaire collects basic time-constant information such as the type of provider, the centre’s proportion of children from households with low socioeconomic status (SES) and the pedagogical group concept prior to the pandemic. The subsequent weekly questionnaire collects information about the current week and contains time-varying variables such as the number and age of children currently attending the ECEC centre, the current application of certain protective measures as well as the number of children, staff and parents who were tested positive for COVID-19 (laboratory confirmed infections). Recent studies show that data on infections from the ECEC centre registry better reflects infection pressure than official data from the local public health departments [14].

In CW22/2021 the questionnaire was extended to include questions on the use of protective measures not covered before, such as wearing face masks by staff and regularly using Covid-19 rapid tests for staff and children, as well as vaccination of staff. The analysis presented here includes data from CW26/2021 to CW14/2022, thus covering two phases (a,b) of the 4th (Delta) wave (4a: CW26/2021-CW38/2021, 4b: CW39/2021-CW51/2021) and the upslope of the 5th (Omicron) wave (CW01/2022-12/2022). This classification is oriented on the proposed RKI retrospective classification [15], but covers more preceding months, as some lead time is essential for the functioning of the applied panel data models. CW52/2021 is excluded from the data, as most ECEC centres had only a limited offer.

### Dependent Variables

We use the occurrence of and the numbers of reported laboratory confirmed infections in children, in parents and in pedagogical staff per week as dependent variables. ECEC centre managers were asked to document any new laboratory confirmed cases of COVID-19. Infections were reported separately for staff members, parents and children. Due to data protection regulations, detailed information on infections in staff was only asked in ECEC centres with at least seven staff members (which applies to 97% of our sample). We assume a mean serial interval between two Delta and Omicron infections of 4 days, and 5.2 to 6.5 days when incubation period is added [16]. The average time lag from the onset of illness in an infectious case to the onset of illness of a case infected by that case takes 5-6 days, and a further 1-3 days are assumed after the result of a laboratory diagnosis is available at the district health department [17]. To reflect this time delay, the dependent variable is estimated with a one week lead. Hence, our models estimate the occurrence/rate of infections in CW X+1 with data from CW X.

### Independent Variables

#### Time-constant variables

*Socioeconomic status (SES):* COVID-19 infections are well known to follow a social gradient [18–20]. To control for social composition, ECEC managers were asked to estimate the proportion of children from families with low SES on a 4-point Likert scale (i.e. below 10% children with low SES background, 11% to 30%, 31% to 60%, or above 60%, respectively).

#### Time-varying variables

*Protective measures:* In every week, managers specified which protective measures were actually implemented. We include information about pharmaceutical as well as on non-pharmaceutical measures. Considering pharmaceutical measures, we include the vaccination coverage among staff members, expressed as the percentage of employees who have received at least one vaccination. For wave 5, we could further utilise information on the so-called booster coverage among staff, hence the percentage of employees who have received at least 3 shots. Considering non-pharmaceutical measures, we include regular wearing of a face mask by staff outside the pedagogical setting, e.g. in contact with parents and colleagues, regular wearing a face mask by staff within his own group, i.e. when children were present. Further the regular ventilation of the rooms, if groups were separated indoors and if staff and children were tested regularly for SARS-CoV-2 infection with rapid tests is included in the model.

*Infections in children, parents and staff:* As we are also interested in the direction of transmission between children, parents and staff, we further include the number of other infections in the model, e.g. when our model estimates the number of infections in children in CW X+1 as dependent variable, we include the number of infections in parents and staff in week X as within and between effect. Since we have no data on direct contacts, infection routes and processes, conclusions can only be drawn from the chronological sequence of infections.

#### Controls

*Local Covid-19 incidence at district level:* To control for the level of the local pandemic we include the weekly incidence of COVID-19 by district as reported by the mandatory surveillance system (number of newly reported cases within seven days in a population of 100,000 as provided by the RKI). To further control for local trends we further include in the model the change of the 7-day incidence on district level within one week. Thus, we do not only control for the local level of the pandemic, but for local trends, too.

*Number of children:* Information on how many children aged 0-2, 3-6, and 7 years and older attended the ECEC centre was provided by ECEC managers. These numbers changed weekly due to (regional) holidays, regional outbreaks and measures taken on federal state or district level.

We use both variables, local infection rate and number of children to quantify exposure to the virus. We do not control the number of employees, as this is proportional to the number of children in care.

### Statistical analysis

We use *time-constant* variables related to the centre (known from the baseline questionnaire, e.g. SES), average differences in the time-varying variables *between* ECEC centres and further changes *within* a centre from one week to another. Since infections are still a rather rare occurrence in the centres, our dependent variable contains too many zeros to correspond to a common count data distribution. Therefore, we utilise a two-step design oriented at a hurdle approach [21] to handle the problem of excess zeros/zero inflation in our data. This approach allows us to distinguish between processes that (1) lead to an infection in a centre in the first place, hence the occurrence of any infections and processes that (2) concern the spread within a centre, hence the number of infections in those ECEC centres where any infections occur in the observation period. Doing so, we estimate the overall likelihood of the occurrence of an infection within the period of observation with a logit-model with a dichotomous dependent variable (any infection vs. no infection) in all centres. Subsequently, we estimate the number of infections in all centres in which any infections occur at all.^1^ Both steps are applied in all the corresponding periods, hence in wave 4a, 4b or 5. Doing so, we use models with a (1) binomial and (2) a negative binomial distribution with quadratic parameterization [22]. For both processes, we apply a random-effect panel model with demeaned data. This allows us to estimate the effects of time-constant variables, between-unit differences and within-unit changes at the same time [23, 24].

1 $$\begin{aligned} P(Y_{i,t+1}|X_{i,t}) = \frac{\text {exp}(\alpha + \beta _{1within}(x_{it}-\bar{x}_i)+\beta _{2between}\bar{x}_i+\beta _{3}z_i+\upsilon _{i1}+\upsilon _{1t})}{1 + \text {exp}(\alpha + \beta _{1within}(x_{it}-\bar{x}_i)+\beta _{2between}\bar{x}_i+\beta _{3}z_i+\upsilon _{i1}+\upsilon _{1t})} \end{aligned}$$

2 $$\begin{aligned} log(E(y_{i,t+1})) = \alpha + \beta _{1within}(x_{it}-\bar{x}_i)+\beta _{2between}\bar{x}_i+\beta _{3}z_i+\upsilon _{i1}+\upsilon _{1t} \end{aligned}$$

We specify both our models with a leading y variable (t+1). $\beta _1$ estimates within-units effects, hence what happens in the next weeks after an ECEC centre decides for example to require staff to wear face masks when in contact with children of their group. $\beta _2$ estimates between-units effects, hence the average effect of e.g. wearing face masks when in contact with children or the average staff vaccination rate. Since the chronological order of events is not taken into account here, these effects are prone to endogeneity and can only be interpreted with great caution, as all time unit-time-points where the preventive rule is applied are compared to all unit-time-points where the rule is not applied. $\beta _3$ estimates effects of time-constant variables, such as SES. $\upsilon _{i1}$ and $\upsilon _{1t}$ are unit- and time-fixed effects. Exponential coefficients of both models could be interpreted as odds ratios (OR) or incidence rate ratios (IRR), i.e. how much the expected probability (formula 1) or the expected count (formula 2) changes multiplicatively when x increases by one. We include a district’s weekly 7-day incidence and the weekly number of children currently attending the centre as a control variable. Lastly, we added the weekly change rate of the district COVID-19 incidence as an additional variable to control for local trends. All control variables are included as within and between effects.

We excluded several variables from the multivariate regression analysis because they show strong correlations with others or were rather rarely applied at all. First, we excluded surface disinfection, as preceding analyses [2] did not show any substantial results. Second, our data includes a number of questions aimed at contact restrictions, namely group separation indoors as well as outdoors and a fixed assignment of staff to certain groups (See Fig. 3). Although it would be plausible to at least consider the separation of the children groups and the fixed assignment of staff to groups separately, further analysis showed that those measures are highly correlated.^2^ To reduce multicollinearity, we include only the variable for group separation indoors as a proxy for general contact restrictions in our models. Third, we exclude the information on regular temperature measurement for children and staff, as both protective measures are used very rarely and never have been recommended in the German context and the variables are highly correlated ($$.58^{***}$$) as well. We provide further details on variable selection and a brief discussion of the excluded variables in the Appendix.

## Results

### Infections in ECEC Centres over time and the dissemination of variants of concern of the COVID-19 Virus

**Fig. 1:**
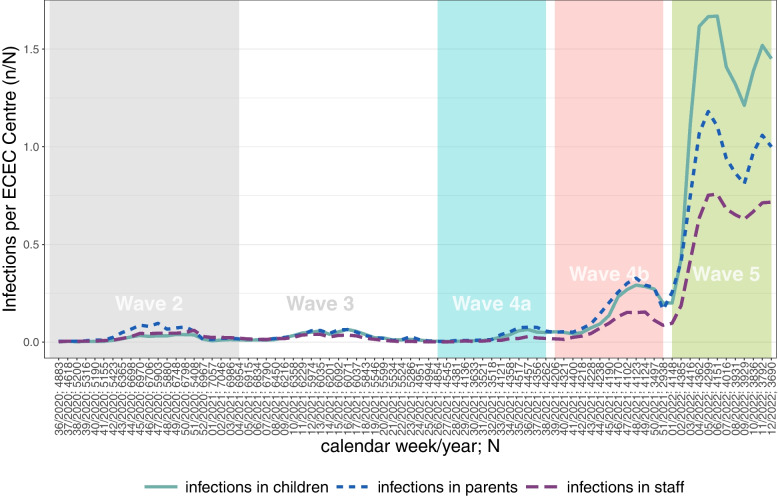
Sum of infection n per ECEC centres N over time per wave. Source: ECEC centre registry, average number of infections (n) in children, parents and staff in ECEC centres (N) per week, (n/N per week), own calculations

Figure 1 shows the development of COVID-19 infections in children, parents and staff between CW36/2020 and CW12/2022 per calendar week. The different waves of the pandemic are highlighted with colored/shaded boxes. The grey area on the left side marks wave 2, the white area marks wave 3. The 4th wave is separated into 2 areas, 4a, 4b, and the 5th wave. Wave 4a marks the beginning of the 4th wave in late summer and autumn 2021. The overall course here is very similar to the preceding waves 2 and 3. Wave 4b, i.e. the development in late fall and before Christmas 2021 is characterised by a significant increase in COVID-19 incidence compared to the previous waves. Finally, wave 5 is characterised by a further significant increase in COVID-19 incidence of all types compared to the previous waves exceeding anything seen before in the pandemic.

**Fig. 2:**
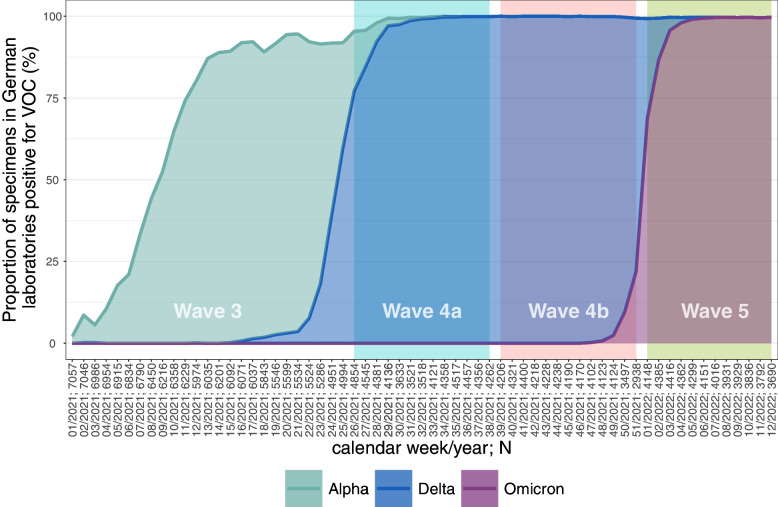
Dissemination of variants of concern of the COVID-19 Virus. Source: https://www.rki.de/DE/Content/InfAZ/N/Neuartiges_Coronavirus/Daten/VOC_VOI_Tabelle.xlsx?__blob=publicationFile, proportion of specimens in German laboratories positive for different VOC (%), own calculations

Figure 2 describes the latest dissemination of COVID-19 variants of concern in the 2 phases of the 4th wave. While the so-called delta variant was the most widespread form in both periods, wave 4a and 4b, 5 is dominated by the so-called Omicron variant.

### Protective measures over time

**Fig. 3:**
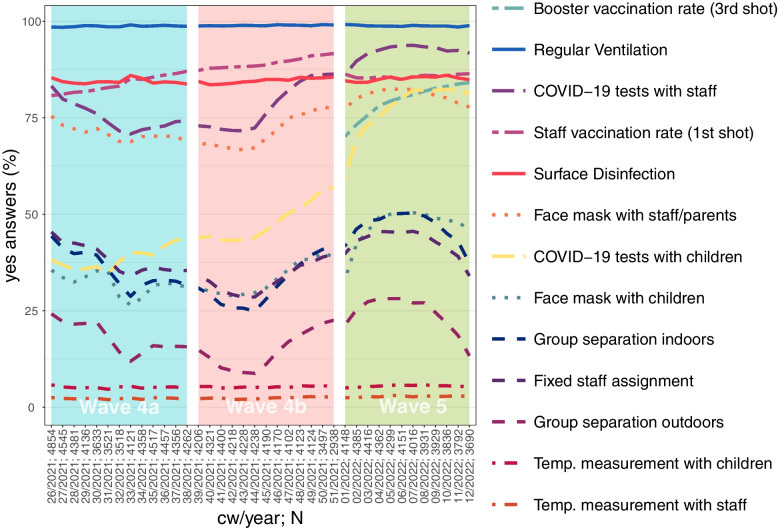
Protective measures in ECEC centres over time. Source: ECEC centre registry, share (%) of ECEC centres which implement certain protective measures, own calculations

Figure 3 shows the development of the application of certain protective measures over time. Measures which were conceptually similar are shown in the same line type, e.g. regular temperature measurement for children and staff are both shown in long dashed lines. Overall, we can observe a dip in most variables in the summer months 2021, and an increase since the middle of wave 4b. Regular ventilation was applied by almost all centres at a very high level with almost no variation over time; the same holds true for surface disinfection, although the level was lower. Almost no variation could also be observed in regular temperature measurement of children and staff - both were only used by a very small number of ECEC centres. Since the vaccination of the staff started well before the survey was conducted, staff vaccination rate starts on a high level in CW26/2021 and increases constantly thereafter. In CW01/2022, we can observe a drop to a lower level. This is due to the fact that the questionnaire, in particular the vaccination question, was revised again at the turn of the year 2021/22, including an additional question for the rate of staff who had received the 3rd shot (booster vaccination). The latter rises from 70% in CW01/2022 to 84% in CW12/2022. Substantially high levels were also observed in the use of COVID-19 rapid tests of staff and in the use of face masks of staff outside the own group setting. Less than 50% of centres used COVID-19 rapid tests of children regularly in CW26/2021. As the summer 2021 progressed, the availability of tests continued to increase and these were carried out increasingly frequently. Nevertheless, a decline in the usage of face masks can be observed from the middle of the 5th wave onwards.

With respect to the variables related to keeping distance, we found indoor group separation and fixed staff assignment to follow a very similar path while outdoor group separation remained less frequent. The high correlation of the individual items can already be seen here in the Fig. 3. Distance measures tended to be suspended in the summer of 2021, but increased sharply again in the fall. Here, too, however, there was a clear decline from the middle of the 5th wave onward. Due to the high correlation and/or the low frequency of certain measures, not all were included in the multivariate models (See Chapter 2.4). Further, we did not include the regular temperature measurement indicators at all, as the rate of implementation was very low. With group separation indoors, we only include one distance variable to avoid bias from correlated explanatory variables (See Fig. A2 and A3). Overall, the correlations of the protective measures seemed very stable between waves and weeks. We further do not include surface disinfection, as it has not shown any convincing results in other studies [2].

### Factors associated with COVID-19 infections in ECEC centres

**Fig. 4:**
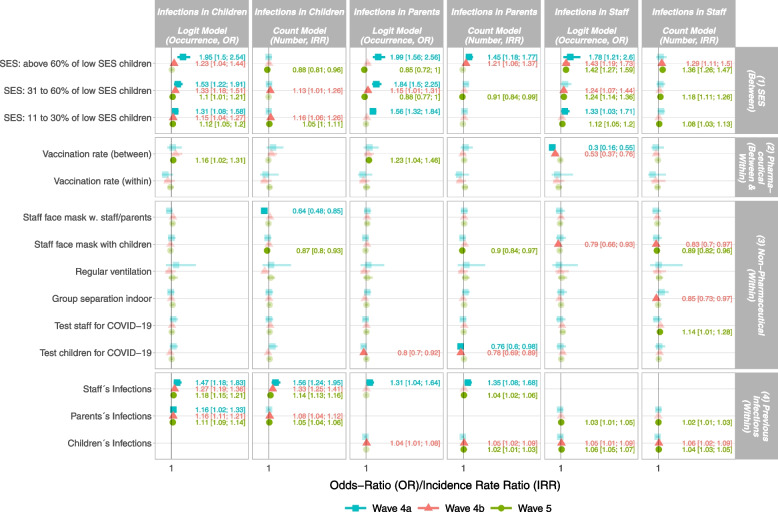
SARS-CoV-2 infections in children, parents and staff. Effects from a hurdle approach for wave 4a, 4b and 5. Source: ECEC centre registry, estimates from logit and count models for SARS-CoV-2 infections children, parents and staff. Odds ratios (OR, logit Models) and incidence rate ratios (IRR, count models), significant coefficients ($p < 0.05$) are printed in opaque with OR/IRR and 95% CI, non significant coefficients are printed in transparent colours. Full models in Tables A1, A2 and A3, own calculations

Figure 4 shows selected coefficients from our 6 multivariate models as forest plot and odds ratios (OR)/incidence rate ratios (ICC) with 95% confidence intervals (CI). The first two columns show the coefficients from a model with infections in children as dependent variable, hence, the first column shows coefficients from the logit model with a dichotomized dependent variable (any infections in children vs. no infections in children (formula 1), the second column shows the coefficients from the count model (formula 2). Coefficients are shown as OR (column 1, 3, 5, logit models) and IRR (column 2, 4, 6, count models). Hence, the coefficients in the first column could be read as multiplicative effects on the odds to observe any infection (occurrence), and the coefficients of the second column as multiplicative effects on the rate, hence the number of infections expected (number). Every column contains coefficients for all waves. The wave is indicated by colour and shape of the point estimate, a cyan square refers to wave 4a, a red triangle refers to wave 4b and a green dot refers to wave 5. Significant coefficients ($p < 0.05$) are printed in opaque with OR/IRR and 95% CI, non significant coefficients are printed in transparent colours. Effects for the dependent variable “infections in parents” and “infections in staff” could be found in the columns 3 and 4 as well as in 5 and 6, respectively.

The vertical stratification of Fig. 4 is by type in four categories, as indicated by the grey boxes on the right. The first 3 rows contain between effect estimates for (1) SES, the following two rows contain between and within effects for pharmaceutical measures, hence for vaccination (2), and the next 6 rows contain the within effect estimates for non-pharmaceutical protective measures like wearing face masks or ventilation (3). The last 3 rows contain the within effects of infections (4). The models underlying Fig. 4 including all (within and between) effect estimates can be found in the Appendix in Table A1 for the wave 4a, Table A2 for wave 4b and Table A3 for wave 5.

### Results from multivariate regression analysis

#### Effects of SES

We find significant effects of the SES on the occurrence and number of all types of infections. Considering the occurrence and number of infections in children (column 1 and 2 in Fig. 4), the strongest effects are found in the model for occurrence (column 1 in Fig. 4) in wave 4a (blue square, OR 1.95 [1.5; 2.54], 95 % confidence intervals in parenthesis). When compared to ECEC centres with a share of 0 to 10 % of children from low SES households (reference group), ECEC centres with 60 % or more children from low SES households were nearly twice as likely to report an infection with children in wave 4a. We found this effect decreasing in size with the increasing frequency of infections in wave 4b (red triangle, OR 1.23 [1.04; 1.44]) and disappearing in wave 5 (green dot, n.s., printed in transparent colours). The same decreasing pattern is also found for the other SES levels in column 1 in Fig. 4. For the number of infections in children (column 2 in Fig. 4), SES does not play a role in wave 4a, but in 4b, and wave 5, but the effects are rather small and do not follow a clear pattern, e.g. the IRR is 1.16 [1.06; 1.26] for centres with a share of 10 to 30 % of children from low SES households in wave 4b, meaning that in those centres, the expected number of infections is 1.16 times greater than in centres with a share of 0 to 10 %. For ECEC centres with above 60 % of children from low SES households, we do find a small negative effect (IRR 0.88 [0.81; 0.96]) in wave 5, hence the expected number of infections is 0.88 times lower compared to the reference group.

For the occurrence of infections in parents (column 3 in Fig. 4), SES plays a large role in wave 4a, a small one in wave 4b, and even a significantly negative role in wave 5. For the number of infections in parents, we found SES to play a role, but only in centres with above 60% of children from low SES households, and the role is decreasing with the ongoing of the pandemic. We found a significant, but small negative effect in wave 5, too, for ECEC centres with above 31 to 60 % of children from low SES households. For the occurrence of infections in staff, we find a rather strong SES effect in wave 4a and decreasing effects in wave 4b and wave 5. Regarding the number of infections in staff, we observe an increasing SES effect over time. While there was no significant SES effect on the number of infections in staff in wave 4a and only a significant in centres with above 60% of children from low SES households in wave 4b, the number of infections in staff in wave 5, hence, with omicron, follows a clear SES pattern. Overall, we find that the main effects of SES concern the occurrence, and not so much the number of infections. Note that these are comparisons between centres and do not take into account the chronological order of events and are therefore susceptible to endogeneity. However, since there is no temporal change in SES as it is a one-time assessment prior to the pandemic, this is not problematic in this case.

#### Effects of pharmaceutical protective measures

The staff’s vaccination quote is a pharmaceutical intervention and differs from SES in that there is no dichotomous coding here, but rather the percentage (coded 0 to 1, where 1 equals 100%) of vaccinated pedagogical staff in the centre is asked for on a weekly basis. The vaccination rate therefore differs between centres as well as within centres over time. However, and in contrast to the following dichotomously coded variables, only a gradual increase (and no absolute change) is possible here. Considering the between effects of vaccination against infection, we find it to be positively associated with COVID-19-infections among children and parents in wave 5, but clearly negative when it comes to occurrence of infections in staff. The former apparently stems from endogeneity, hence, it is reasonable that infections among children or parents do not stem from a higher vaccination rate among staff, but rather that, e.g., increased (reports of) infections among children or parents make staff more likely to become vaccinated. Otherwise, it is also possible that higher vaccination rates in the overall population (or among staff) might lead to a false sense of security.

Conversely, there is a clear negative relationship between a high staff vaccination rate and the occurrence of infections among staff in wave 4a (OR 0.3 [0.16; 0.55]),^3^ and less so, in wave 4b (OR 0.53 [0.37; 0.76]). We found this relation to disappear (OR 0.95, [0.84; 1.07, n.s.]) in wave 5 with the emergence of the Omicron variant.

We further rerun the models for wave 5 exchanging the vaccination rate with the booster vaccination rate (See Table A4, and using both, i.e. vaccination rate and booster rate in one model, see Table A5). Doing so, we find significant positive between-effects between the occurrence and number of infections in children and parents and a higher booster rate. When comparing the effect sizes between the first shot vaccination rate and the booster vaccination rate (See Table A5), the effect sizes in the booster rate tend to be larger in all models, but still, considering infections in staff, they fail to reach statistical significance.

We do not find any significant within effects in any model for the vaccination rate, the only exception here is a significant negative within effect of an increasing booster vaccination rate on the number of infections in parents in wave 5, see Tables A4 and A5.

### Effects of non-pharmaceutical protective measures

Overall, we expect the introduction of a protective measure within an ECEC centre to reduce the number of infections. An exception here is the introduction of regular testing, which might have a positive effect, as testing is not a direct protective measure, but rather a detection procedure. We find that the introduction of staff wearing face masks when in contact with other staff or parents, hence outside the group setting, reduces the number of infections in children in wave 4a (IRR 0.64 [0.48; 0.85]). The introduction of staff wearing face masks within the group setting, hence, in contact with children, significantly reduces the occurrence (OR 0.79 [0.66; 0.93]) and number (IRR 0.83 [0.7; 0.97]) of infections in staff in wave 4b. Further we found it to reduce the number of infections in all count models, hence in the number of infections in children, parents and staff in wave 5. We found no significant effect of regular ventilation in any model. Further, we found the introduction of indoor group separation to significantly reduce the number of infections in staff in wave 4b (IRR 0.85 [0.73; 0.97]), but not to have any effects in the other models. We found the introduction of regular testing of staff to increase the number of reported infections in staff in wave 5 (IRR 1.14 [1.01; 1.28]). Hence, regular testing of pedagogical staff results in fewer undetected infections here. Considering the introduction of tests for children, we found them to significantly reduce the occurrence and number of infections in parents in wave 4a and 4b, but not in wave 5.

#### Within effects of infections

Regarding the question who infects whom, i.e. chains of infection, we found significant effects on the occurrence and number of infections in children with both, previous infections in staff and in parents. The strongest effects on infections in children are found due to previous infections in staff in both models, hence for occurence (OR 1.47 [1.18; 1.83]) and number (IRR 1.56 [1.24; 1.95]), in wave 4a, while these effects are decreasing with increasing frequency of infections in wave 4b and 5. Infections in parents play a smaller, but still significant role here. Considering the occurrence and number of infections in parents, we found that previous infections in staff are followed by an increased occurrence and number of infections in parents in wave 4a. Further, previous infections in staff have a small positive effect on the number of infections in parents in wave 5. Previous infections in children only have small effects on the occurrence and number of infections in parents in wave 4b, and we found a small but significant effect of infections in children on the number of infections in parents in wave 5. The occurrence and number of infections in staff seems rather unaffected by previous infections in parents and children. We found a small effect of previous infections in parents on both, occurrence and number of infections in staff in wave 5, and further small effects of previous infections in children on the occurrence and number of infections in staff in wave 4b and 5. Overall, the effect size of previous infections seems significantly reduced with the emergence of omicron in wave 5.

## Discussion

Over the course of the Delta and Omicron waves, we found the effects of socio-economic status and vaccination on the risk of infection has decreased, at least when it comes to infections in children and parents, while the preventive effect of masks worn by pedagogical staff has increased. Social status of the families where the children were coming from was a strong indicator in ECEC for an increasing risk of infection both in staff and children during wild-type and Alpha waves [2]. Hypotheses for this association included different living conditions, such as crowded living, and/or different working conditions among socially deprived population groups [25]. The two-step design in this paper sheds some light on the mechanism between SES and infections. Children usually attend an ECEC institution in their neighbourhood, hence SES of the children is a sound indicator of the social composition in the centre’s environment. By far the stronger effects of SES in our analysis are seen in the probability to observe infections in a centre at all, and less in the number. This indicates that infections are more frequently brought in from the environment if the centre is located in a socially deprived area, but social status of the children itself has a rather small effect on the spread of infections through the centre, at least to other children and parents.

Why the effect of SES on infections among staff increases in Omicron waves is open to speculation. One possibility could be that staff in socially deprived areas are vaccinated to a lower degree, as vaccination is known to follow a social gradient [26], and at the same time the vaccine protection against infection with omicron is known to be weaker [27].

The decrease in vaccine effectiveness demonstrates and confirms the good protection of the vaccine against an infection with the Delta variant within the first two months [28, 29]. The lower estimated effectiveness of the vaccine against SARS-CoV-2 infections in the (Delta) wave 4b, in December 2021, again is supported by findings from other studies [28–30] who showed a decline of effectiveness after 6 months between 10 and 30% percentage points. In wave 5, we found that the protective effect against infections has almost disappeared which agrees again with findings from several other authors who found consistently lower efficacy of the vaccine against Omicron infections compared with Delta infections when the same number of vaccinations, the same vaccine types and the same time period after vaccination was analysed [27, 28, 31].

Possible explanations for the increased risk to observe an infection in parents and children in ECEC centers with higher vaccination rates among staff in the (Omicron) wave 5 could be related to endogeneity, i.e. when reports of high local infection rates encourage staff to get vaccinated. They also might be explained by a sense of false security [32, 33], e.g. when perceived protection through increasingly widespread (booster) vaccination leads to a lower perception of risk of infection and corresponding behavioural changes in the general population. If the latter assumption holds true, then the lack of between effects between vaccination rates and infections among staff suggests that while preventive behavior may be declining in the general population, protection of staff from infections in day care centers remains a high priority despite increasingly available (booster) vaccinations.

Considering the significant negative within effect of an increasing booster rate on the number of infections in parents found in Tables A4 and A5, we can only assume that there is either a temporal concurrence between a decrease in general local infections and an increasing availability of booster vaccines for staff or a marginal protective effect from the vaccination itself, preventing the transmission of infections from staff to parents. As we find all within effects of the (booster) vaccination rates to be at least negatively associated (effects are negative, but not sigificant) with the number and occurence of infections in staff, a protective effect is quite likely, but our models, however, fail to provide significant evidence.

Our analysis of non-pharmaceutical protective measures identified the most conclusive protective effect for the use of face masks by the staff. In wave 4a, the introduction of face masks worn by staff when in contact with other staff or parents lowers the number of infections in children only, staff starting to wear face masks in contact with children lowers the occurence and number of infections in staff in wave 4b. Further, staff starting to wear face masks in contact with children lowers the number of infections in children, parents and staff in wave 5. Thus, in the presence of Omicron, wearing a mask effectively reduces the number of infections in all models, successfully preventing secondary infections in the ECEC centre. Here, the two-step estimation designs demonstrates: the introduction of face masks does not help to prevent the occurrence of infections in ECEC centres, but they have a clear effect on the number of infections, thus limiting transmission.

Overall, our results confirm previous research on the effectiveness of face masks [11], but not all studies on the topic find significant effects for infections in staff [12]. Our study provides clear longitudinal evidence for the protective effect on staff. However, recent studies suggest that staff wearing face masks impair emotion reading [34] and can even negatively affect the quality of interaction between staff and children [35]. Thus, their use in contact with children must be carefully weighed against the educational adverse effects.

We do not find an effect for ventilation, but this is probably due to the very high adherence rate (See Fig. 3). We only find a small effect for the introduction of indoor group separation on the number of infections in staff in wave 4b. While in our first study we saw that contact reduction between groups has been effective [2], it seems to play a minor role in the face of increasing vaccination rates and the widespread use of face masks.

Due to the strong correlation between the contact reduction variables mentioned above in Chapter 2.4, further analysis (See Appendix Figs. A7 and A8) showed mixed results. Since the vast majority of significant effects for contact restrictions are clearly negative in all model variants tested, we consider group separation to be beneficial to some extent, but without being able to specify the exact range of effects.

Considering detection methods, serial testing of pedagogical staff increased the number of (detected) infections in staff, hence regular testing results in fewer undetected infections in wave 5. On the other hand, serial testing of children seems to offer some protection against infections in parents, but only in wave 4a and 4b. Here it could be hypothesised that serial tests - which were carried out by the parents - might have increased the general sensitivity of the parents with regard to hygiene. Considering the other detection method in our data, temperature measurement on children, in-depth additional analyses (See Appendix Fig. A8) provides some hints that the introduction of temperature measurement in children might reduce the occurrence and number of infections in children, but the underlying estimates are based on comparatively very few observations and biased by correlation with the other temperature measurement variable and should be interpreted with great caution, if at all. Here, additional research is needed, presumably from areas where these measures have been applied more frequently.

Our results on possible chains of infections indicate that infections of staff had been the main drivers for infections in children and, less so, for infections in parents (only in wave 4a), but this effect is decreasing from (Delta) waves 4a and 4b to (Omicron) wave 5, perhaps due to increasing vaccination rates and the increased use of masks. In addition, the force of infection in the whole society which has increased in the same sequence may have led to coincidental infections that had nothing to do with exposure in the ECEC centre. Previous infections in parents clearly can be attributed to be followed by infections in children in all waves, but the effect size is rather small. Further, we found significant effects of preceding infections in parents on infections in staff in wave 5. Previous infections in children seem to play a significant, but smaller role for both, infections in parents and staff.

The significance of this effect, particularly in relation to preceding infections among staff, increases over time and is particularly pronounced in wave 5. Thus, previous infections of children in particular become more threatening to staff. Although the role of children as true primary cases may be substantially underestimated [5] in our “ecological” analysis, we can conclude that staff infections from children are playing an increasingly important role. This, in turn, could be related to increasing contagiousness in society at large, or it could be that children actually become more contagious when infected with Omicron compared to Delta.

## Limitations

As a general limitation, we acknowledge that a management’s decision to implement certain protective measures in their ECEC centre (and the corresponding notification in our questionnaires) is not always followed and put into daily practice by all employees. There were no precise instructions on our side given to the participating managers when exactly a protective measure is considered implemented (e.g., information on the frequency of a particular measure, for example, we do not know whether tests for SARS-CoV-2 were applied once a day or only once a week or what kind of rapid tests where used). However, detailed hygiene plans with descriptions of the procedures were handed out by the health offices. Data on staff vaccination status were also voluntary and represent only the level of knowledge of the management of the ECEC and may not reflect the precise vaccination rate. In addition, the question about SES was asked retrospectively and is therefore susceptible to recall bias, which is particularly common in COVID-19 studies [36]. Due to the special circumstances of the pandemic, many of our survey instruments are also new designs that went into the field immediately and for which extensive pretesting was hardly possible. The regular weekly survey also made it necessary to keep the scope and thus the depth of focus of the survey rather low, so as not to place an undue burden on the ECEC managers. However, all these aspects apply to many COVID-19 studies [37].

## Conclusions

Our analyses show that as infections increase and the omicron variant becomes more widespread, the protective effect of vaccination against infection decreases significantly. Protective measures, especially the use of face masks are becoming increasingly important, as the omicron variant is proving to be particularly sensitive to protective measures. At the same time, the well-known SES effect loses its importance for infections in children and parents, but remains important for infections in staff. Our results further show that overall the risk of infection from staff tends to decrease, but the risk of infection from parents and children increases. Vaccination against SARS-CoV-2 has shown good effectiveness against severe course of disease, however, it is not very effective against transmission of the Omicron variant. It is therefore prudent to strengthen usage of non-pharmaceutical protective measures like face masks to reduce introduction of the virus. In particular, staff in ECEC centres with a high proportion of children from low SES backgrounds should act with particular caution and wear masks sooner than others, with the goal to keep ECEC centres open as long as possible to avoid reactive closure.

## Supplementary Information

**Additional file 1.**

## Data Availability

Anonymized data will be passed on to external researchers after the project has been finalised (presumably January 2023), provided that they are used for the purpose of scientific secondary and subsequent use and under the conditions of the DJI Research Data Centre (incl. deletion periods, thematic limitation, citation, etc.). The anonymised survey data and research results are stored for 10 years as proof of good scientific practice and in accordance with the funding regulations. Please contact the first author (fneuberger@dji.de) to obtain the data (only possible after official release of the data, presumably January 2023).

## Abbreviations

CW: Calendar week
DJI: German Youth Institute (Deutsches Jugendinstitut)
IRR: Incidence rate ratio
OR: Odds ratio
REWB: Random-effect-within-between
RKI: Robert Koch Institute
SES: Socioeconomic status
VOC: Variant of concern

## Footnotes

1 See Fig. A1 in the Appendix for details on the distribution. Figure A1 shows the distribution of the dependent variables in the individual waves with and without the selection by the two-step approach.

2 See Figs. A2 and A3 for correlation plots for all waves. Correlation is $$.62^{***}$$ between fixed staff assignment and indoor group separation, $$.45^{***}$$ between staff assignment and outdoor group separation, $$.56^{***}$$ between group separation indoors and outdoors.

3 Note that this OR refers to a comparison between a 0 and a 100 % rate. E.g., in wave 4a, a 10% increase in the vaccination rate would lead to an OR of .89. As the vaccination rate is coded 0 to 1, where 1 equals 100%, we can calculate the OR for a 10% difference by multiplying the log odds of the between effect, which is -1.22 (See column 5 in Table A1) by 0.1, leading to a OR of $exp(-1.22*0.1)=0.89$, hence an increase by 10% would lead to a 0.89 times lower chance to report an infection in staff.
