# Supplementary material for: Delta and Omicron: protective measures and SARS-CoV-2 infections in day care centres in Germany in the 4th and 5th wave of the pandemic 2021/2022
Source: BMC Public Health. 2022 Nov 17;22:2106. doi: 10.1186/s12889-022-14521-x (PMC9670080; doi:10.1186/s12889-022-14521-x)
Supplement: Supplementary file 1 — Additional file 1. [file 12889_2022_14521_MOESM1_ESM.pdf]

Appendix

Delta and Omicron: Protective measures and SARS-CoV-2 infections in day care centres in Germany in the 4th and 5th wave of the pandemic 2021/2022

Franz Neuberger, Mariana Grgic, Udo Buchholz, Hanna Lena Maly-Motta, Sina Fackler, Ann-Sophie Lehfeld, Walter Haas, Bernhard Kalicki, and Susanne Kuger

November 15, 2022

Table A1: Logit and count models for infections in children, parents and staff, wave 4a

	(1): Child, 4a occurrence	(2): Child, 4a. number	(3): Parent, 4a. occurrence	(4): Parent, 4a. number	(5): Staff, 4a occurrence	(6): Staff, 4a number
Intercept	−4.68*	−1.42	−4.88*	−2.95*	−3.98*	−1.05
	[−6.15; −3.21]	[−3.31; 0.47]	[−6.31; −3.44]	[−4.41; −1.49]	[−5.87; −2.09]	[−3.22; 1.11]
SES: 11 to 30% of low SES children (ref.: below 10%)	0.27*	−0.00	0.44*	0.08	0.28*	0.20
	[0.08; 0.46]	[−0.18; 0.18]	[0.28; 0.61]	[−0.08; 0.23]	[0.03; 0.54]	[−0.05; 0.44]
SES: 31 to 60% of low SES children	0.42*	0.02	0.61*	0.17	0.29	0.15
	[0.20; 0.65]	[−0.18; 0.23]	[0.41; 0.81]	[−0.00; 0.35]	[−0.03; 0.62]	[−0.14; 0.45]
SES: above 60% of low SES children	0.67*	−0.01	0.69*	0.37*	0.57*	0.17
	[0.40; 0.93]	[−0.24; 0.22]	[0.44; 0.94]	[0.17; 0.57]	[0.19; 0.96]	[−0.18; 0.52]
Regular ventilation of rooms (between)	−0.92	−1.12	−0.52	0.52	−0.95	−1.14
	[−2.30; 0.46]	[−2.94; 0.71]	[−1.84; 0.80]	[−0.82; 1.85]	[−2.76; 0.86]	[−3.18; 0.91]
Regular ventilation of rooms (within)	0.32	0.17	0.16	0.21	−0.16	0.03
	[−0.55; 1.20]	[−0.69; 1.03]	[−0.59; 0.90]	[−0.61; 1.02]	[−1.19; 0.87]	[−1.08; 1.13]
Group separation indoor (between)	0.11	0.09	0.07	−0.05	0.25	−0.12
	[−0.08; 0.30]	[−0.09; 0.27]	[−0.11; 0.24]	[−0.21; 0.10]	[−0.02; 0.51]	[−0.39; 0.15]
Group separation indoor (within)	−0.06	0.01	0.08	0.15	0.01	0.24
	[−0.34; 0.23]	[−0.28; 0.31]	[−0.17; 0.34]	[−0.11; 0.41]	[−0.38; 0.39]	[−0.13; 0.61]
Staff face mask with staff/parents (between)	0.21	−0.08	0.26*	0.05	0.01	−0.04
	[−0.03; 0.46]	[−0.32; 0.16]	[0.04; 0.48]	[−0.15; 0.24]	[−0.32; 0.33]	[−0.37; 0.29]
Staff face mask with staff/parents (within)	−0.20	−0.45*	0.09	0.05	−0.13	−0.22
	[−0.49; 0.09]	[−0.74; −0.16]	[−0.17; 0.34]	[−0.21; 0.31]	[−0.52; 0.27]	[−0.64; 0.21]
Staff face mask with children (between)	0.10	0.03	0.12	0.04	0.22	0.20
	[−0.09; 0.30]	[−0.16; 0.23]	[−0.05; 0.30]	[−0.12; 0.20]	[−0.05; 0.49]	[−0.08; 0.47]
Staff face mask with children (within)	−0.00	−0.10	−0.07	−0.10	−0.07	−0.16
	[−0.29; 0.29]	[−0.39; 0.19]	[−0.32; 0.19]	[−0.36; 0.16]	[−0.46; 0.33]	[−0.57; 0.25]
Test staff for COVID-19 (between)	0.04	0.18	−0.06	0.09	0.06	−0.21
	[−0.21; 0.29]	[−0.06; 0.41]	[−0.28; 0.16]	[−0.10; 0.28]	[−0.29; 0.41]	[−0.54; 0.11]
Test staff for COVID-19 (within)	0.15	0.03	−0.05	−0.05	−0.08	−0.14
	[−0.14; 0.43]	[−0.26; 0.31]	[−0.30; 0.19]	[−0.30; 0.20]	[−0.47; 0.31]	[−0.54; 0.26]
Test children for COVID-19 (between)	0.12	−0.00	−0.03	−0.02	0.13	−0.07
	[−0.07; 0.31]	[−0.19; 0.18]	[−0.20; 0.14]	[−0.17; 0.13]	[−0.14; 0.39]	[−0.33; 0.19]
Test children for COVID-19 (within)	0.15	0.26	−0.23	−0.27*	−0.04	0.07
	[−0.14; 0.43]	[−0.02; 0.54]	[−0.49; 0.02]	[−0.52; −0.02]	[−0.42; 0.35]	[−0.31; 0.45]
Vaccination rate (between)	0.10	0.30	0.24	0.15	−1.22*	−0.22
	[−0.40; 0.61]	[−0.19; 0.79]	[−0.22; 0.70]	[−0.27; 0.57]	[−1.83; −0.60]	[−0.83; 0.38]
Vaccination rate (within)	−0.68	−0.31	−0.35	−0.43	−0.35	−0.62
	[−1.50; 0.14]	[−1.20; 0.59]	[−1.10; 0.40]	[−1.23; 0.37]	[−1.39; 0.69]	[−1.73; 0.49]
Nr. of Infections: Staff (between)	2.78*	1.01*	0.36	0.03		
	[1.88; 3.68]	[0.38; 1.65]	[−0.59; 1.31]	[−0.61; 0.67]		
Nr. of Infections: Staff (within)	0.39*	0.44*	0.27*	0.30*		
	[0.17; 0.60]	[0.22; 0.67]	[0.04; 0.49]	[0.08; 0.52]		
Nr. of Infections: Parents (between)	3.10*	0.82*			−0.02	0.25
	[2.68; 3.51]	[0.59; 1.05]			[−0.68; 0.63]	[−0.22; 0.71]
Nr. of Infections: Parents (within)	0.15*	0.02			−0.07	0.01
	[0.02; 0.28]	[−0.09; 0.13]			[−0.32; 0.18]	[−0.21; 0.24]
7 day Incidence (within)	0.01*	0.01*	0.01*	0.01*	0.02*	0.01*
	[0.01; 0.02]	[0.01; 0.01]	[0.01; 0.01]	[0.01; 0.01]	[0.01; 0.02]	[0.00; 0.01]
7 day Incidence (between)	0.00*	−0.00	0.00*	−0.00	0.01	−0.00
	[0.00; 0.01]	[−0.01; 0.00]	[0.00; 0.01]	[−0.00; 0.00]	[−0.00; 0.01]	[−0.01; 0.01]
Increase 7 day Incidence (t to t+x, within)	0.00*	0.01*	0.00*	0.01*	0.00*	0.01*
	[0.00; 0.01]	[0.00; 0.01]	[0.00; 0.00]	[0.00; 0.01]	[0.00; 0.01]	[0.00; 0.01]
Increase 7 day Incidence (t to t+x, between)	0.00*	0.01*	0.00*	0.01*	0.00*	−0.00
	[0.00; 0.01]	[0.00; 0.01]	[0.00; 0.00]	[0.00; 0.01]	[0.00; 0.00]	[−0.02; 0.01]
Nr. of Infections: Children (between)			3.40*	1.21*	1.96*	0.75*
			[2.95; 3.85]	[0.94; 1.48]	[1.21; 2.71]	[0.25; 1.26]
Nr. of Infections: Children (within)			−0.07	−0.06	−0.05	−0.06
			[−0.18; 0.05]	[−0.17; 0.05]	[−0.25; 0.15]	[−0.24; 0.12]
AIC	7711.23	5815.29	9764.54	7915.29	4829.54	2788.09
Log Likelihood	−3821.61	−2872.65	−4848.27	−3922.65	−2380.77	−1359.04
Num. obs.	36472	5716	36472	7448	36472	3071
Num. groups: week	13	13	13	13	13	13
Num. groups: token	4721	655	4721	851	4721	352
Var: week (Intercept)	0.17	0.29	0.43	0.31	0.03	0.42
Var: token (Intercept)	0.96	0.00	1.05	0.00	1.80	0.00

$^{*}p < 0.05$, All estimates as log odds, own calculations.

Table A2: Logit and count models for infections in children, parents and staff, wave 4b

	(1): Child, 4b occurrence	(2): Child, 4b. number	(3): Parent, 4b. occurrence	(4): Parent, 4b. number	(5): Staff, 4b occurrence	(6): Staff, 4b number
Intercept	−3.91*	−2.74*	−3.25*	−2.16*	−2.57*	−1.97*
	[−4.75; −3.06]	[−3.61; −1.87]	[−4.08; −2.41]	[−2.82; −1.50]	[−3.44; −1.71]	[−2.69; −1.26]
SES: 11 to 30% of low SES children (ref.: below 10%)	0.14*	0.15*	0.06	0.06	0.08	0.04
	[0.04; 0.24]	[0.06; 0.23]	[−0.04; 0.16]	[−0.01; 0.14]	[−0.03; 0.20]	[−0.05; 0.13]
SES: 31 to 60% of low SES children	0.29*	0.12*	0.14*	0.05	0.21*	0.11
	[0.16; 0.41]	[0.01; 0.24]	[0.01; 0.27]	[−0.04; 0.15]	[0.06; 0.36]	[−0.01; 0.23]
SES: above 60% of low SES children	0.20*	0.02	0.11	0.19*	0.36*	0.26*
	[0.04; 0.37]	[−0.13; 0.17]	[−0.05; 0.28]	[0.06; 0.31]	[0.17; 0.55]	[0.11; 0.41]
Regular ventilation of rooms (between)	−0.17	0.44	−0.23	0.17	−1.09*	−0.11
	[−0.91; 0.58]	[−0.35; 1.23]	[−0.95; 0.49]	[−0.39; 0.73]	[−1.84; −0.35]	[−0.70; 0.49]
Regular ventilation of rooms (within)	−0.19	−0.41	−0.15	−0.13	−0.04	−0.05
	[−0.67; 0.29]	[−0.88; 0.06]	[−0.58; 0.28]	[−0.55; 0.30]	[−0.58; 0.49]	[−0.59; 0.49]
Group separation indoor (between)	0.12*	0.11*	0.00	0.02	0.11	−0.07
	[0.01; 0.23]	[0.01; 0.21]	[−0.11; 0.11]	[−0.06; 0.11]	[−0.02; 0.24]	[−0.17; 0.04]
Group separation indoor (within)	−0.01	0.02	−0.11	−0.04	−0.11	−0.17*
	[−0.15; 0.12]	[−0.11; 0.15]	[−0.23; 0.01]	[−0.15; 0.07]	[−0.26; 0.04]	[−0.31; −0.03]
Staff face mask with staff/parents (between)	0.10	−0.04	0.09	0.03	0.15*	0.04
	[−0.02; 0.22]	[−0.15; 0.07]	[−0.02; 0.21]	[−0.06; 0.12]	[0.01; 0.29]	[−0.08; 0.15]
Staff face mask with staff/parents (within)	0.13	0.10	0.08	0.05	0.00	−0.09
	[−0.03; 0.28]	[−0.04; 0.25]	[−0.06; 0.21]	[−0.07; 0.18]	[−0.17; 0.17]	[−0.25; 0.07]
Staff face mask with children (between)	0.05	0.03	0.02	0.04	−0.02	−0.03
	[−0.06; 0.15]	[−0.07; 0.13]	[−0.08; 0.13]	[−0.05; 0.12]	[−0.15; 0.10]	[−0.14; 0.08]
Staff face mask with children (within)	−0.07	0.02	0.07	0.10	−0.24*	−0.19*
	[−0.22; 0.07]	[−0.12; 0.16]	[−0.06; 0.21]	[−0.02; 0.22]	[−0.41; −0.07]	[−0.35; −0.03]
Test staff for COVID-19 (between)	0.24*	0.16*	0.12	0.03	0.13	0.10
	[0.09; 0.38]	[0.03; 0.30]	[−0.02; 0.26]	[−0.08; 0.14]	[−0.04; 0.29]	[−0.05; 0.25]
Test staff for COVID-19 (within)	0.05	0.12	0.03	0.07	0.09	0.16
	[−0.10; 0.20]	[−0.03; 0.27]	[−0.10; 0.16]	[−0.06; 0.19]	[−0.08; 0.27]	[−0.01; 0.33]
Test children for COVID-19 (between)	0.08	−0.02	−0.03	−0.07	−0.02	0.08
	[−0.02; 0.18]	[−0.11; 0.07]	[−0.13; 0.07]	[−0.15; 0.01]	[−0.14; 0.10]	[−0.02; 0.17]
Test children for COVID-19 (within)	−0.11	−0.06	−0.22*	−0.25*	0.13	0.11
	[−0.26; 0.05]	[−0.21; 0.09]	[−0.36; −0.08]	[−0.37; −0.12]	[−0.04; 0.31]	[−0.05; 0.27]
Vaccination rate (between)	0.30	0.21	0.16	−0.07	−0.64*	−0.20
	[−0.04; 0.64]	[−0.10; 0.51]	[−0.18; 0.49]	[−0.33; 0.18]	[−1.01; −0.27]	[−0.49; 0.10]
Vaccination rate (within)	−0.35	−0.43	−0.26	−0.33	−0.39	−0.24
	[−0.92; 0.22]	[−1.06; 0.20]	[−0.78; 0.25]	[−0.84; 0.18]	[−1.02; 0.24]	[−0.93; 0.45]
Nr. of Infections: Staff (between)	1.18*	1.01*	0.04	0.18*		
	[0.99; 1.36]	[0.87; 1.15]	[−0.18; 0.26]	[0.00; 0.35]		
Nr. of Infections: Staff (within)	0.24*	0.28*	0.03	0.04		
	[0.17; 0.31]	[0.22; 0.35]	[−0.04; 0.10]	[−0.02; 0.10]		
Nr. of Infections: Parents (between)	1.25*	0.64*			0.34*	0.09
	[1.14; 1.37]	[0.55; 0.72]			[0.20; 0.48]	[−0.01; 0.19]
Nr. of Infections: Parents (within)	0.14*	0.08*			0.02	0.00
	[0.10; 0.19]	[0.04; 0.12]			[−0.03; 0.07]	[−0.04; 0.05]
7 day Incidence (within)	0.00*	0.00*	0.00*	0.00*	0.00*	0.00*
	[0.00; 0.00]	[0.00; 0.00]	[0.00; 0.00]	[0.00; 0.00]	[0.00; 0.00]	[0.00; 0.00]
7 day Incidence (between)	0.00	−0.00	0.00*	0.00*	0.00*	0.00
	[−0.00; 0.00]	[−0.00; 0.00]	[0.00; 0.00]	[0.00; 0.00]	[0.00; 0.00]	[−0.00; 0.00]
Increase 7 day Incidence (t to t+x, within)	0.00*	0.00*	0.00*	0.00*	0.00*	0.00*
	[0.00; 0.00]	[0.00; 0.00]	[0.00; 0.00]	[0.00; 0.00]	[0.00; 0.00]	[0.00; 0.00]
Increase 7 day Incidence (t to t+x, between)	0.00*	0.00*	0.00*	0.00*	0.00*	0.00*
	[0.00; 0.00]	[0.00; 0.00]	[0.00; 0.00]	[0.00; 0.00]	[0.00; 0.00]	[0.00; 0.00]
Nr. of Infections: Children (between)			0.78*	0.69*	0.83*	0.59*
			[0.66; 0.91]	[0.60; 0.79]	[0.71; 0.96]	[0.51; 0.67]
Nr. of Infections: Children (within)			0.04*	0.05*	0.05*	0.05*
			[0.01; 0.08]	[0.02; 0.08]	[0.01; 0.09]	[0.02; 0.09]
AIC	23398.54	27466.35	28499.27	34979.47	19354.41	18541.44
Log Likelihood	−11665.27	−13698.17	−14215.64	−17454.74	−9643.21	−9235.72
Num. obs.	40133	20183	40133	23826	40133	15756
Num. groups: week	13	13	13	13	13	13
Num. groups: token	4283	1999	4283	2326	4283	1558
Var: week (Intercept)	0.14	0.12	0.21	0.14	0.21	0.24
Var: token (Intercept)	0.44	0.10	0.70	0.09	0.68	0.02

$^{*}p < 0.05$, All estimates as log odds, own calculations.

Table A3: Logit and count models for infections in children, parents and staff, wave 5

	(1): Child, 5 occurrence	(2): Child, 5. number	(3): Parent, 5. occurrence	(4): Parent, 5. number	(5): Staff, 5 occurrence	(6): Staff, 5 number
Intercept	−2.56*	−1.16*	−1.93*	−1.01*	−1.96*	−1.36*
	[−3.13; −1.99]	[−1.61; −0.70]	[−2.70; −1.17]	[−1.51; −0.52]	[−2.51; −1.41]	[−1.77; −0.94]
SES: 11 to 30% of low SES children (ref.: below 10%)	0.11*	0.05*	−0.01	−0.05	0.12*	0.08*
	[0.05; 0.18]	[0.00; 0.10]	[−0.10; 0.09]	[−0.11; 0.00]	[0.05; 0.18]	[0.03; 0.13]
SES: 31 to 60% of low SES children	0.10*	−0.01	−0.13*	−0.09*	0.22*	0.16*
	[0.01; 0.19]	[−0.08; 0.06]	[−0.26; −0.00]	[−0.17; −0.01]	[0.13; 0.31]	[0.10; 0.23]
SES: above 60% of low SES children	0.04	−0.13*	−0.16*	−0.07	0.35*	0.31*
	[−0.08; 0.16]	[−0.21; −0.04]	[−0.33; −0.00]	[−0.17; 0.03]	[0.24; 0.46]	[0.23; 0.39]
Regular ventilation of rooms (between)	0.47	0.36	0.61	0.28	−0.04	0.16
	[−0.05; 1.00]	[−0.05; 0.78]	[−0.09; 1.32]	[−0.17; 0.73]	[−0.53; 0.45]	[−0.21; 0.53]
Regular ventilation of rooms (within)	0.08	0.15	0.09	0.14	0.07	0.06
	[−0.25; 0.42]	[−0.11; 0.41]	[−0.26; 0.43]	[−0.11; 0.40]	[−0.27; 0.42]	[−0.20; 0.33]
Group separation indoor (between)	0.10*	0.01	0.05	−0.00	0.02	−0.07*
	[0.03; 0.17]	[−0.04; 0.07]	[−0.05; 0.15]	[−0.06; 0.06]	[−0.05; 0.09]	[−0.12; −0.02]
Group separation indoor (within)	0.10	0.07	0.03	0.05	0.02	−0.01
	[−0.01; 0.20]	[−0.01; 0.15]	[−0.08; 0.14]	[−0.02; 0.13]	[−0.09; 0.12]	[−0.10; 0.07]
Staff face mask with staff/parents (between)	0.02	−0.03	0.08	0.06	0.18*	0.11*
	[−0.07; 0.12]	[−0.10; 0.04]	[−0.05; 0.21]	[−0.02; 0.14]	[0.09; 0.28]	[0.04; 0.18]
Staff face mask with staff/parents (within)	0.09	−0.03	0.08	0.05	−0.07	−0.08
	[−0.04; 0.22]	[−0.12; 0.07]	[−0.05; 0.22]	[−0.04; 0.15]	[−0.20; 0.06]	[−0.18; 0.02]
Staff face mask with children (between)	0.09*	0.08*	0.08	0.08*	−0.07	−0.12*
	[0.02; 0.16]	[0.03; 0.13]	[−0.02; 0.18]	[0.02; 0.14]	[−0.14; 0.00]	[−0.17; −0.07]
Staff face mask with children (within)	−0.08	−0.14*	−0.05	−0.11*	−0.10	−0.12*
	[−0.18; 0.02]	[−0.22; −0.07]	[−0.15; 0.06]	[−0.18; −0.03]	[−0.21; 0.00]	[−0.20; −0.04]
Test staff for COVID-19 (between)	0.11	0.09	0.10	0.04	0.05	−0.06
	[−0.03; 0.26]	[−0.02; 0.20]	[−0.11; 0.30]	[−0.09; 0.17]	[−0.10; 0.19]	[−0.16; 0.04]
Test staff for COVID-19 (within)	0.03	0.07	0.02	0.02	0.14	0.13*
	[−0.11; 0.18]	[−0.03; 0.18]	[−0.13; 0.17]	[−0.09; 0.12]	[−0.01; 0.29]	[0.01; 0.24]
Test children for COVID-19 (between)	0.14*	0.04	0.05	0.03	0.08	0.02
	[0.05; 0.23]	[−0.03; 0.11]	[−0.07; 0.18]	[−0.05; 0.11]	[−0.01; 0.17]	[−0.05; 0.08]
Test children for COVID-19 (within)	0.03	0.07	0.02	0.04	0.03	0.04
	[−0.08; 0.13]	[−0.00; 0.15]	[−0.08; 0.13]	[−0.04; 0.11]	[−0.08; 0.14]	[−0.04; 0.13]
Vaccination rate (between)	0.14*	−0.03	0.21*	0.05	−0.05	−0.03
	[0.02; 0.27]	[−0.12; 0.06]	[0.04; 0.38]	[−0.05; 0.16]	[−0.17; 0.07]	[−0.12; 0.05]
Vaccination rate (within)	−0.09	0.06	0.02	0.11	−0.21	−0.06
	[−0.34; 0.17]	[−0.13; 0.24]	[−0.23; 0.28]	[−0.08; 0.30]	[−0.47; 0.06]	[−0.27; 0.14]
Nr. of Infections: Staff (between)	0.62*	0.59*	0.13*	0.15*		
	[0.56; 0.68]	[0.55; 0.63]	[0.04; 0.22]	[0.09; 0.20]		
Nr. of Infections: Staff (within)	0.17*	0.13*	0.01	0.04*		
	[0.14; 0.19]	[0.12; 0.15]	[−0.01; 0.04]	[0.02; 0.06]		
Nr. of Infections: Parents (between)	0.37*	0.18*			0.13*	0.04*
	[0.33; 0.40]	[0.16; 0.21]			[0.10; 0.16]	[0.02; 0.06]
Nr. of Infections: Parents (within)	0.11*	0.05*			0.03*	0.02*
	[0.09; 0.13]	[0.04; 0.06]			[0.01; 0.04]	[0.01; 0.03]
7 day Incidence (within)	0.00*	0.00*	0.00*	0.00*	0.00*	0.00*
	[0.00; 0.00]	[0.00; 0.00]	[0.00; 0.00]	[0.00; 0.00]	[0.00; 0.00]	[0.00; 0.00]
7 day Incidence (between)	0.00*	−0.00	0.00	−0.00	0.00*	0.00
	[0.00; 0.00]	[−0.00; 0.00]	[−0.00; 0.00]	[−0.00; 0.00]	[0.00; 0.00]	[−0.00; 0.00]
Increase 7 day Incidence (t to t+x, within)	0.00*	0.00*	0.00*	0.00*	0.00*	0.00*
	[0.00; 0.00]	[0.00; 0.00]	[0.00; 0.00]	[0.00; 0.00]	[0.00; 0.00]	[0.00; 0.00]
Increase 7 day Incidence (t to t+x, between)	0.00*	0.00*	−0.00	0.00*	0.00*	0.00*
	[0.00; 0.00]	[0.00; 0.00]	[−0.00; 0.00]	[0.00; 0.00]	[0.00; 0.00]	[0.00; 0.00]
Nr. of Infections: Children (between)			0.16*	0.21*	0.24*	0.23*
			[0.12; 0.20]	[0.18; 0.23]	[0.21; 0.27]	[0.22; 0.25]
Nr. of Infections: Children (within)			0.01	0.02*	0.06*	0.04*
			[−0.00; 0.02]	[0.01; 0.03]	[0.05; 0.07]	[0.03; 0.05]
AIC	38714.80	94839.17	39371.94	82143.44	38009.20	68245.68
Log Likelihood	−19323.40	−47384.59	−19651.97	−41036.72	−18970.60	−34087.84
Num. obs.	31030	30037	31030	28498	31030	29430
Num. groups: week	11	11	11	11	11	11
Num. groups: token	4073	3821	4073	3566	4073	3683
Var: week (Intercept)	0.03	0.04	0.03	0.02	0.05	0.04
Var: token (Intercept)	0.24	0.12	0.96	0.25	0.19	0.04

$^*p < 0.05$, All estimates as log odds, own calculations.

Table A4: Replication of Table A3, but including estimates for the rate of staff with booster vaccinations (3rd shot) instead of 1st shot vaccination rate

	(1): Child, 5 occurrence	(2): Child, 5. number	(3): Parent, 5. occurrence	(4): Parent, 5. number	(5): Staff, 5 occurrence	(6): Staff, 5 number
Intercept	−2.70*	−1.32*	−2.18*	−1.32*	−2.04*	−1.36*
	[−3.27; −2.13]	[−1.77; −0.87]	[−2.92; −1.43]	[−1.81; −0.84]	[−2.59; −1.50]	[−1.77; −0.95]
SES: 11 to 30% of low SES children (ref.: below 10%)	0.11*	0.05*	−0.01	−0.04	0.11*	0.08*
	[0.05; 0.18]	[0.00; 0.10]	[−0.10; 0.08]	[−0.10; 0.01]	[0.05; 0.18]	[0.03; 0.12]
SES: 31 to 60% of low SES children	0.09*	−0.02	−0.14*	−0.09*	0.21*	0.16*
	[0.00; 0.18]	[−0.09; 0.04]	[−0.26; −0.01]	[−0.17; −0.01]	[0.12; 0.30]	[0.10; 0.22]
SES: above 60% of low SES children	0.05	−0.12*	−0.17*	−0.06	0.34*	0.30*
	[−0.07; 0.16]	[−0.21; −0.04]	[−0.33; −0.01]	[−0.16; 0.03]	[0.23; 0.46]	[0.23; 0.38]
Regular ventilation of rooms (between)	0.41	0.40*	0.47	0.31	−0.08	0.17
	[−0.10; 0.91]	[0.00; 0.80]	[−0.20; 1.15]	[−0.12; 0.75]	[−0.56; 0.40]	[−0.19; 0.53]
Regular ventilation of rooms (within)	0.18	0.18	0.14	0.16	0.09	0.09
	[−0.15; 0.50]	[−0.08; 0.43]	[−0.20; 0.48]	[−0.09; 0.40]	[−0.25; 0.42]	[−0.17; 0.36]
Group separation indoor (between)	0.13*	0.02	0.07	0.00	0.03	−0.06*
	[0.06; 0.19]	[−0.03; 0.07]	[−0.02; 0.17]	[−0.05; 0.06]	[−0.04; 0.10]	[−0.11; −0.01]
Group separation indoor (within)	0.10	0.08*	0.03	0.06	0.01	−0.02
	[−0.00; 0.21]	[0.01; 0.16]	[−0.07; 0.14]	[−0.02; 0.13]	[−0.10; 0.11]	[−0.09; 0.06]
Staff face mask with staff/parents (between)	0.02	−0.04	0.08	0.06	0.18*	0.11*
	[−0.07; 0.11]	[−0.11; 0.03]	[−0.05; 0.21]	[−0.01; 0.14]	[0.09; 0.28]	[0.04; 0.18]
Staff face mask with staff/parents (within)	0.08	−0.02	0.06	0.04	−0.09	−0.07
	[−0.04; 0.21]	[−0.11; 0.07]	[−0.06; 0.19]	[−0.05; 0.14]	[−0.21; 0.04]	[−0.17; 0.02]
Staff face mask with children (between)	0.09*	0.08*	0.06	0.07*	−0.06	−0.11*
	[0.02; 0.16]	[0.03; 0.13]	[−0.03; 0.16]	[0.01; 0.13]	[−0.13; 0.01]	[−0.15; −0.06]
Staff face mask with children (within)	−0.08	−0.13*	−0.05	−0.10*	−0.10*	−0.11*
	[−0.18; 0.02]	[−0.21; −0.06]	[−0.15; 0.06]	[−0.17; −0.03]	[−0.21; −0.00]	[−0.19; −0.04]
Test staff for COVID-19 (between)	0.11	0.06	0.13	0.05	0.06	−0.06
	[−0.04; 0.26]	[−0.05; 0.16]	[−0.07; 0.33]	[−0.07; 0.17]	[−0.09; 0.20]	[−0.16; 0.05]
Test staff for COVID-19 (within)	0.06	0.07	0.01	−0.01	0.11	0.11
	[−0.08; 0.21]	[−0.03; 0.18]	[−0.13; 0.16]	[−0.11; 0.09]	[−0.04; 0.26]	[−0.00; 0.22]
Test children for COVID-19 (between)	0.11*	0.04	0.03	0.01	0.06	0.01
	[0.02; 0.20]	[−0.02; 0.11]	[−0.10; 0.15]	[−0.07; 0.09]	[−0.03; 0.15]	[−0.06; 0.07]
Test children for COVID-19 (within)	0.03	0.06	0.00	0.02	0.01	0.03
	[−0.08; 0.13]	[−0.02; 0.14]	[−0.10; 0.11]	[−0.05; 0.10]	[−0.10; 0.11]	[−0.05; 0.11]
Booster Vaccination rate (between)	0.38*	0.12	0.63*	0.34*	0.07	−0.05
	[0.22; 0.54]	[−0.00; 0.23]	[0.41; 0.84]	[0.20; 0.47]	[−0.09; 0.22]	[−0.16; 0.06]
Booster Vaccination rate (within)	−0.19	−0.09	−0.31	−0.25*	−0.18	−0.12
	[−0.50; 0.12]	[−0.32; 0.14]	[−0.62; 0.00]	[−0.48; −0.03]	[−0.50; 0.14]	[−0.37; 0.13]
Nr. of Infections: Staff (between)	0.63*	0.59*	0.14*	0.16*		
	[0.57; 0.69]	[0.55; 0.64]	[0.06; 0.23]	[0.10; 0.21]		
Nr. of Infections: Staff (within)	0.17*	0.13*	0.01	0.04*		
	[0.14; 0.19]	[0.12; 0.15]	[−0.01; 0.04]	[0.02; 0.05]		
Nr. of Infections: Parents (between)	0.37*	0.18*			0.14*	0.05*
	[0.33; 0.40]	[0.16; 0.20]			[0.10; 0.17]	[0.02; 0.07]
Nr. of Infections: Parents (within)	0.11*	0.05*			0.03*	0.02*
	[0.09; 0.13]	[0.04; 0.06]			[0.01; 0.05]	[0.01; 0.03]
7 day Incidence (within)	0.00*	0.00*	0.00*	0.00*	0.00*	0.00*
	[0.00; 0.00]	[0.00; 0.00]	[0.00; 0.00]	[0.00; 0.00]	[0.00; 0.00]	[0.00; 0.00]
7 day Incidence (between)	0.00*	−0.00	0.00	−0.00	0.00*	0.00
	[0.00; 0.00]	[−0.00; 0.00]	[−0.00; 0.00]	[−0.00; 0.00]	[0.00; 0.00]	[−0.00; 0.00]
Increase 7 day Incidence (t to t+x, within)	0.00*	0.00*	0.00*	0.00*	0.00*	0.00*
	[0.00; 0.00]	[0.00; 0.00]	[0.00; 0.00]	[0.00; 0.00]	[0.00; 0.00]	[0.00; 0.00]
Increase 7 day Incidence (t to t+x, between)	0.00*	0.00*	0.00	0.00*	0.00*	0.00*
	[0.00; 0.00]	[0.00; 0.00]	[−0.00; 0.00]	[0.00; 0.00]	[0.00; 0.00]	[0.00; 0.00]
Nr. of Infections: Children (between)			0.15*	0.20*	0.24*	0.23*
			[0.11; 0.19]	[0.18; 0.22]	[0.21; 0.26]	[0.22; 0.25]
Nr. of Infections: Children (within)			0.01	0.02*	0.06*	0.04*
			[−0.00; 0.02]	[0.01; 0.03]	[0.05; 0.07]	[0.03; 0.05]
AIC	40456.22	99021.38	41214.44	86180.14	39762.68	71142.63
Log Likelihood	−20194.11	−49475.69	−20573.22	−43055.07	−19847.34	−35536.32
Num. obs.	32507	31429	32507	29850	32507	30766
Num. groups: week	11	11	11	11	11	11
Num. groups: token	4145	3880	4145	3625	4145	3736
Var: week (Intercept)	0.04	0.04	0.03	0.02	0.05	0.04
Var: token (Intercept)	0.23	0.12	0.94	0.24	0.19	0.04

$^{*}p < 0.05$, All estimates as log odds, own calculations.

Table A5: Replication of Table A3, but including estimates for the rate of staff with booster vaccinations (3rd shot) and rate of staff with 1st shot vaccination rate

	(1): Child, 5 occurrence	(2): Child, 5. number	(3): Parent, 5. occurrence	(4): Parent, 5. number	(5): Staff, 5 occurrence	(6): Staff, 5 number
Intercept	-2.78^{*}	-1.26^{*}	-2.29^{*}	-1.26^{*}	-2.02^{*}	-1.35^{*}
	$[-3.37; -2.19]$	$[-1.73; -0.79]$	$[-3.07; -1.51]$	$[-1.76; -0.75]$	$[-2.59; -1.45]$	$[-1.78; -0.93]$
SES: 11 to 30% of low SES children (ref.: below 10%)	0.12^{*}	0.05^{*}	-0.00	-0.05	0.11^{*}	0.08^{*}
	$[0.05; 0.18]$	$[0.00; 0.10]$	$[-0.09; 0.09]$	$[-0.11; 0.01]$	$[0.05; 0.18]$	$[0.03; 0.13]$
SES: 31 to 60% of low SES children	0.10^{*}	-0.01	-0.12	-0.09^{*}	0.21^{*}	0.16^{*}
	$[0.01; 0.19]$	$[-0.08; 0.06]$	$[-0.25; 0.01]$	$[-0.16; -0.01]$	$[0.12; 0.30]$	$[0.10; 0.23]$
SES: above 60% of low SES children	0.05	-0.12^{*}	-0.14	-0.06	0.34^{*}	0.30^{*}
	$[-0.07; 0.17]$	$[-0.21; -0.04]$	$[-0.31; 0.02]$	$[-0.16; 0.04]$	$[0.23; 0.46]$	$[0.22; 0.38]$
Regular ventilation of rooms (between)	0.40	0.33	0.53	0.27	-0.06	0.17
	$[-0.13; 0.93]$	$[-0.08; 0.75]$	$[-0.18; 1.23]$	$[-0.18; 0.72]$	$[-0.56; 0.44]$	$[-0.20; 0.55]$
Regular ventilation of rooms (within)	0.11	0.14	0.09	0.14	0.09	0.05
	$[-0.23; 0.45]$	$[-0.12; 0.40]$	$[-0.26; 0.44]$	$[-0.12; 0.39]$	$[-0.26; 0.43]$	$[-0.22; 0.32]$
Group separation indoor (between)	0.12^{*}	0.02	0.08	0.01	0.02	-0.06^{*}
	$[0.05; 0.19]$	$[-0.03; 0.08]$	$[-0.02; 0.17]$	$[-0.05; 0.07]$	$[-0.05; 0.09]$	$[-0.11; -0.01]$
Group separation indoor (within)	0.09	0.07	0.02	0.05	0.01	-0.02
	$[-0.02; 0.20]$	$[-0.01; 0.15]$	$[-0.09; 0.13]$	$[-0.03; 0.13]$	$[-0.10; 0.12]$	$[-0.10; 0.06]$
Staff face mask with staff/parents (between)	0.02	-0.03	0.07	0.06	0.18^{*}	0.11^{*}
	$[-0.08; 0.11]$	$[-0.11; 0.04]$	$[-0.06; 0.20]$	$[-0.02; 0.14]$	$[0.08; 0.27]$	$[0.04; 0.18]$
Staff face mask with staff/parents (within)	0.10	-0.02	0.08	0.05	-0.07	-0.06
	$[-0.02; 0.23]$	$[-0.11; 0.08]$	$[-0.05; 0.21]$	$[-0.04; 0.15]$	$[-0.20; 0.07]$	$[-0.16; 0.04]$
Staff face mask with children (between)	0.09^{*}	0.08^{*}	0.08	0.08^{*}	-0.06	-0.11^{*}
	$[0.02; 0.16]$	$[0.03; 0.13]$	$[-0.02; 0.18]$	$[0.02; 0.14]$	$[-0.13; 0.01]$	$[-0.16; -0.06]$
Staff face mask with children (within)	-0.09	-0.15^{*}	-0.04	-0.11^{*}	-0.11^{*}	-0.12^{*}
	$[-0.19; 0.02]$	$[-0.22; -0.07]$	$[-0.15; 0.06]$	$[-0.18; -0.03]$	$[-0.21; -0.00]$	$[-0.20; -0.05]$
Test staff for COVID-19 (between)	0.13	0.10	0.13	0.06	0.06	-0.06
	$[-0.02; 0.29]$	$[-0.01; 0.21]$	$[-0.08; 0.34]$	$[-0.06; 0.19]$	$[-0.09; 0.20]$	$[-0.16; 0.05]$
Test staff for COVID-19 (within)	0.04	0.08	0.02	0.02	0.13	0.12^{*}
	$[-0.11; 0.19]$	$[-0.03; 0.19]$	$[-0.13; 0.18]$	$[-0.08; 0.13]$	$[-0.03; 0.28]$	$[0.01; 0.24]$
Test children for COVID-19 (between)	0.12^{*}	0.04	0.02	0.01	0.07	0.02
	$[0.03; 0.22]$	$[-0.03; 0.11]$	$[-0.11; 0.15]$	$[-0.07; 0.09]$	$[-0.02; 0.16]$	$[-0.05; 0.08]$
Test children for COVID-19 (within)	0.04	0.08^{*}	0.03	0.04	0.03	0.05
	$[-0.07; 0.15]$	$[0.00; 0.16]$	$[-0.08; 0.14]$	$[-0.04; 0.12]$	$[-0.08; 0.14]$	$[-0.04; 0.13]$
Vaccination rate (between)	0.05	-0.07	0.06	-0.03	-0.08	-0.02
	$[-0.08; 0.18]$	$[-0.16; 0.03]$	$[-0.12; 0.23]$	$[-0.14; 0.08]$	$[-0.20; 0.05]$	$[-0.11; 0.07]$
Vaccination rate (within)	-0.07	0.06	0.11	0.18	-0.19	-0.06
	$[-0.33; 0.19]$	$[-0.13; 0.25]$	$[-0.15; 0.38]$	$[-0.02; 0.37]$	$[-0.46; 0.08]$	$[-0.27; 0.14]$
Booster Vaccination rate (between)	0.41^{*}	0.16^{*}	0.64^{*}	0.35^{*}	0.11	-0.04
	$[0.23; 0.58]$	$[0.04; 0.29]$	$[0.41; 0.87]$	$[0.20; 0.50]$	$[-0.06; 0.28]$	$[-0.16; 0.08]$
Booster Vaccination rate (within)	-0.17	-0.05	-0.30	-0.26^{*}	-0.08	-0.07
	$[-0.49; 0.15]$	$[-0.29; 0.19]$	$[-0.62; 0.03]$	$[-0.49; -0.03]$	$[-0.41; 0.25]$	$[-0.32; 0.19]$
Nr. of Infections: Staff (between)	0.62^{*}	0.59^{*}	0.14^{*}	0.15^{*}		
	$[0.56; 0.68]$	$[0.54; 0.63]$	$[0.05; 0.23]$	$[0.10; 0.21]$		
Nr. of Infections: Staff (within)	0.17^{*}	0.14^{*}	0.01	0.04^{*}		
	$[0.14; 0.20]$	$[0.12; 0.15]$	$[-0.01; 0.04]$	$[0.02; 0.06]$		
Nr. of Infections: Parents (between)	0.36^{*}	0.18^{*}			0.13^{*}	0.04^{*}
	$[0.33; 0.40]$	$[0.16; 0.20]$			$[0.10; 0.16]$	$[0.02; 0.06]$
Nr. of Infections: Parents (within)	0.11^{*}	0.05^{*}			0.03^{*}	0.02^{*}
	$[0.09; 0.12]$	$[0.03; 0.06]$			$[0.01; 0.04]$	$[0.01; 0.03]$
7 day Incidence (within)	0.00^{*}	0.00^{*}	0.00^{*}	0.00^{*}	0.00^{*}	0.00^{*}
	$[0.00; 0.00]$	$[0.00; 0.00]$	$[0.00; 0.00]$	$[0.00; 0.00]$	$[0.00; 0.00]$	$[0.00; 0.00]$
7 day Incidence (between)	0.00^{*}	-0.00	0.00	-0.00	0.00^{*}	0.00
	$[0.00; 0.00]$	$[-0.00; 0.00]$	$[-0.00; 0.00]$	$[-0.00; 0.00]$	$[0.00; 0.00]$	$[-0.00; 0.00]$
Increase 7 day Incidence (t to t+x, within)	0.00^{*}	0.00^{*}	0.00^{*}	0.00^{*}	0.00^{*}	0.00^{*}
	$[0.00; 0.00]$	$[0.00; 0.00]$	$[0.00; 0.00]$	$[0.00; 0.00]$	$[0.00; 0.00]$	$[0.00; 0.00]$
Increase 7 day Incidence (t to t+x, between)	0.00^{*}	0.00^{*}	0.00	0.00^{*}	0.00^{*}	0.00^{*}
	$[0.00; 0.00]$	$[0.00; 0.00]$	$[-0.00; 0.00]$	$[0.00; 0.00]$	$[0.00; 0.00]$	$[0.00; 0.00]$
Nr. of Infections: Children (between)			0.15^{*}	0.20^{*}	0.24^{*}	0.23^{*}
			$[0.11; 0.19]$	$[0.18; 0.23]$	$[0.21; 0.27]$	$[0.22; 0.25]$
Nr. of Infections: Children (within)			0.01	0.02^{*}	0.06^{*}	0.04^{*}
			$[-0.00; 0.02]$	$[0.01; 0.03]$	$[0.04; 0.07]$	$[0.03; 0.05]$
AIC	38281.54	93976.41	38970.24	81478.13	37641.83	67589.92
Log Likelihood	-19104.77	-46951.20	-19449.12	-40702.06	-18784.91	-33757.96
Num. obs.	30700	29726	30700	28233	30700	29132
Num. groups: week	11	11	11	11	11	11
Num. groups: token	4041	3795	4041	3544	4041	3658
Var: week (Intercept)	0.03	0.04	0.03	0.02	0.05	0.04
Var: token (Intercept)	0.23	0.12	0.94	0.24	0.19	0.04

$^{*}p < 0.05$, All estimates as log odds, own calculations.

Figure A1: Visualization of the excess zeros in the data used for two-step approach: Number of infections in (a) children, (b) parents and (c) staff reported per week per ECEC centre per wave

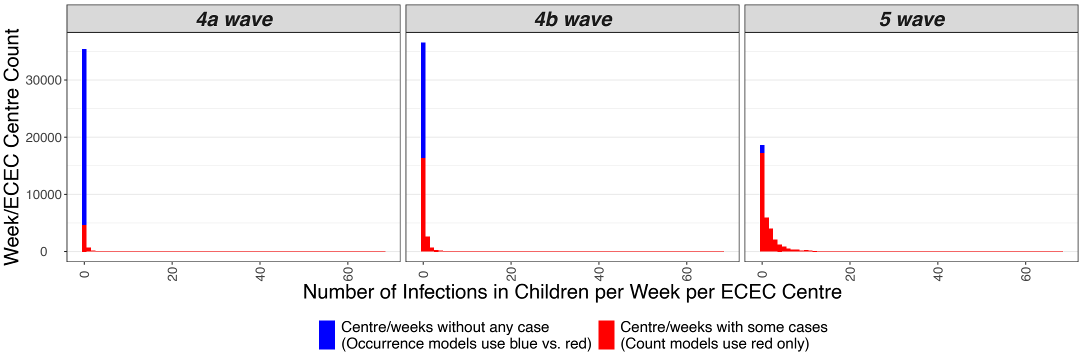

(a) Number of infections per week in children

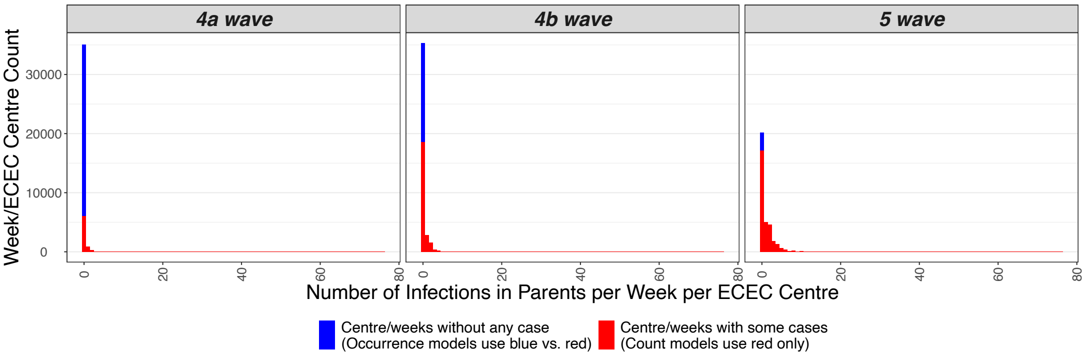

(b) Number of infections per week in parents

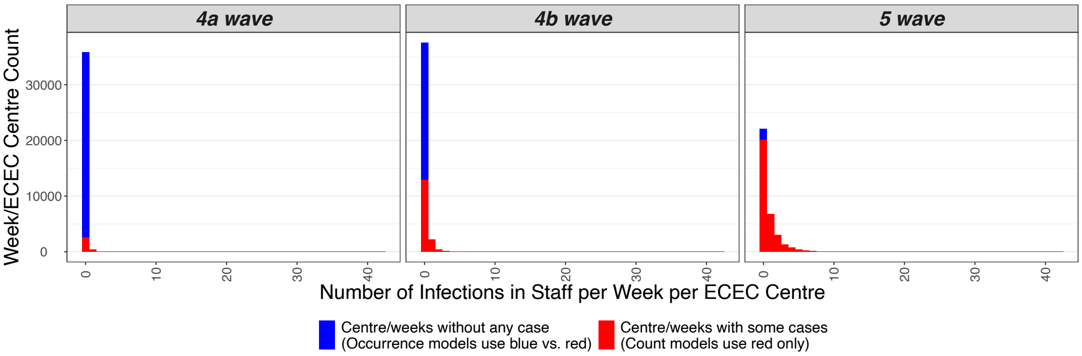

(c) Number of infections per week in staff

Visualization of the excess zeros we lose due to the two-step process. Subfigures show histogramms of the number of infection reported per week in (a) children, (b) parents and (c) staff for the three waves under study. Weeks from ECEC centres which do not report any infections in children, parents or staff in the corresponding wave are highlighted in blue, reported number of infections per week from ECEC centres which do report any infections in children, parents or staff in the corresponding wave are highlighted in red. Source: ECEC centre registry, own calculations.

Figure A2: Correlations of protective measures over waves

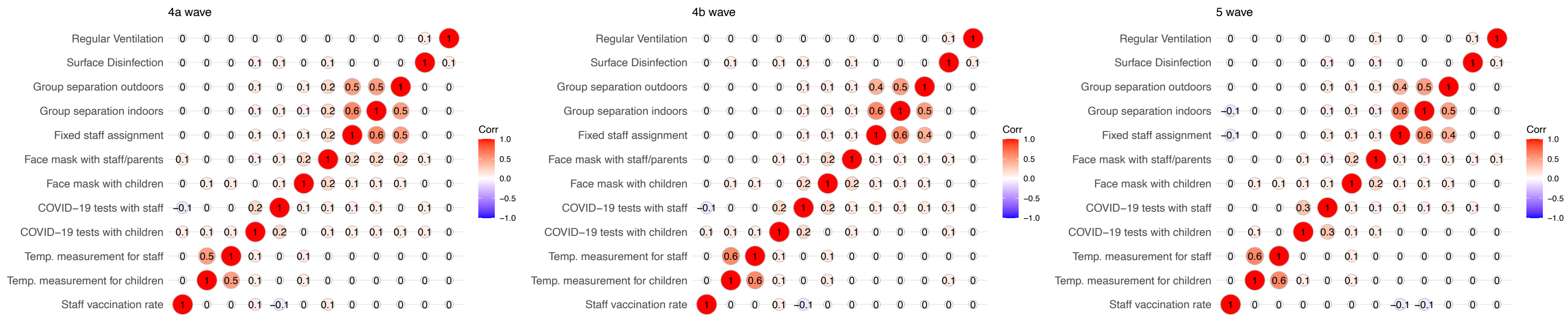

Source: ECEC centre registry, correlation of protective measures, see also Figure 3, own calculations.

Figure A3: Correlations of protective measures over weeks

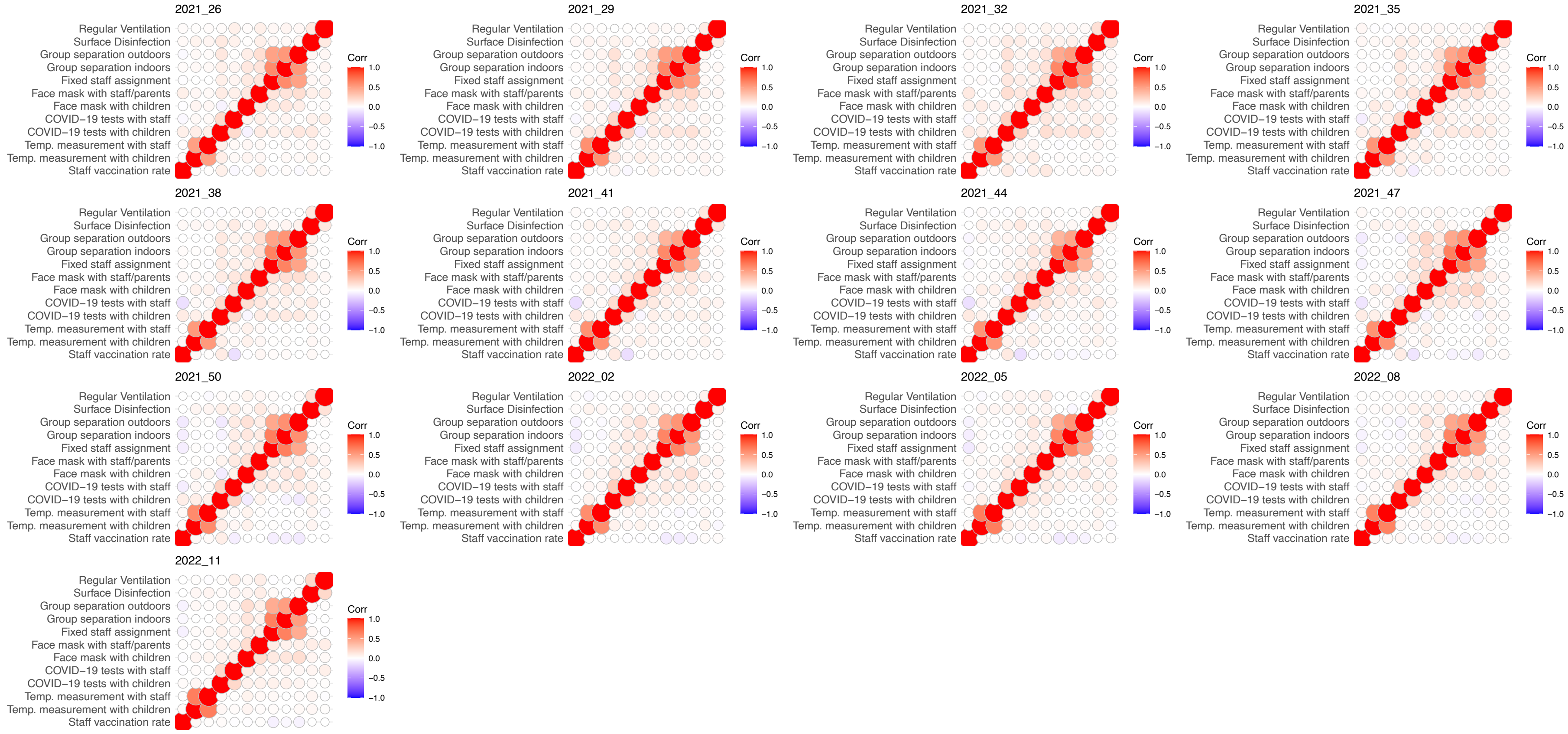

Source: ECEC centre registry, correlation of protective measures for certain weeks (every 3rd week only), see also Figure 3, own calculations.

Figure A4: Within variance of protective measures in wave 4a

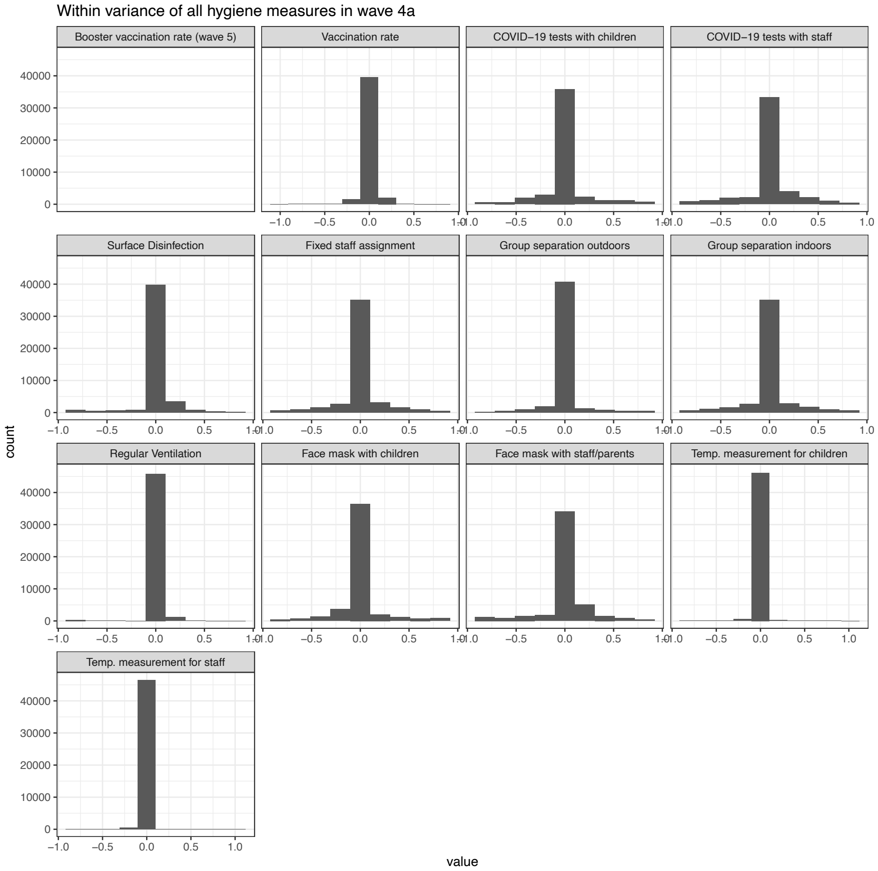

Source: ECEC centre registry, own calculations.

Figure A5: Within variance of protective measures in wave 4b

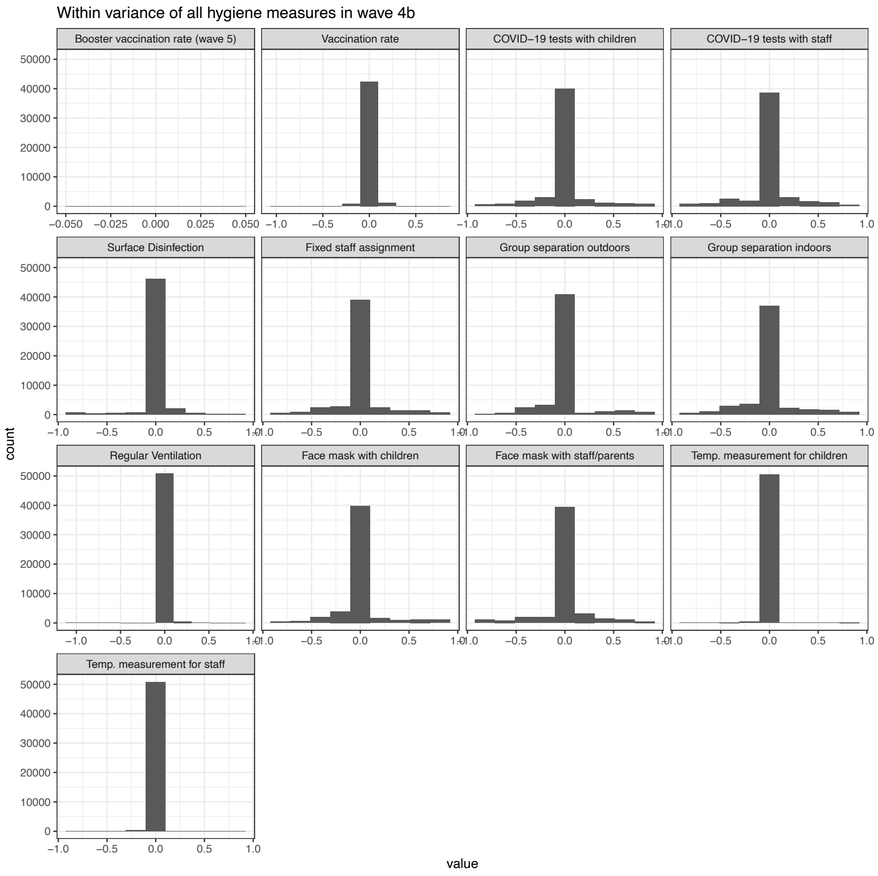

Source: ECEC centre registry, own calculations.

Figure A6: Within variance of protective measures in wave 5

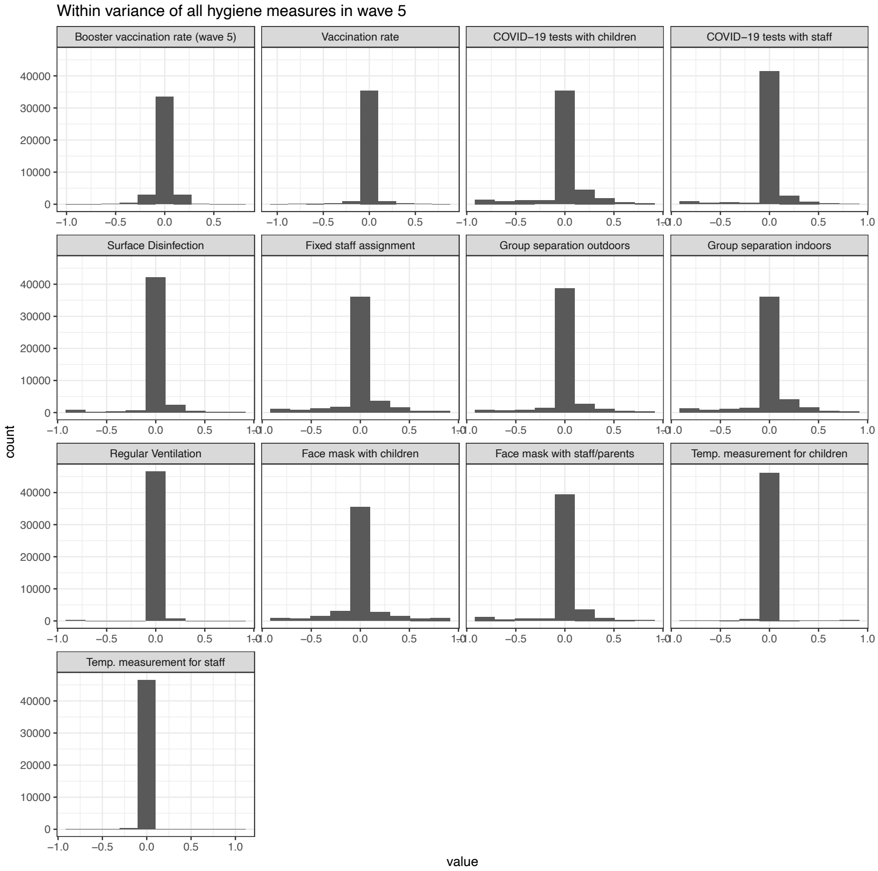

Source: ECEC centre registry, own calculations.

Further Analysis: In-depth analysis and selection of hygiene variables

As mentioned above, we excluded several hygiene measures from the main analysis, namely surface disinfection, group separation outdoors, fixed staff assignment and temperature measurement for staff and children. In the following, we provide a brief discussion of the whys and wherefores of this decision and provide an in-depth analysis of the excluded items. Doing so, we replicate our analyses in different settings. In the first setting, we use summed indices for certain hygiene measures (index setting, see Figure A7), e.g. for testing and contact reduction, hence a more parsemoneous model. In the second setting, we include all individual hygiene measurement variables (See Figure A8), hence, we use a more generous approach (generous setting). The variable selection in the model presented in the paper is based on considerations from both approaches. In the following, we provide a brief discussion of both approaches.

- Appendix Figure A7 is a replication of Figure 4 using sum indices for different groups of hygiene measeres, cumulative effects of similar measures are assumed here (index setting). Here, single dichotomous hygiene items are summed up by thematic groups as described below. The idea behind that approach was to get rid of the high correlations between some items, especially between the contact variables. Appendix Figure A2 provides correlation coefficients for all hygiene measurements for all waves, showing that group separation indoors has a .6 correlation with fixed staff assignment and a .5 correlation with group separation outdoors in all waves, while group separation outdoors only has a .4 correlation with fixed staff assignment in wave 4b and 5. For face masks and tests it goes up to .2, in wave 5 correlation for Covid-19 tests went up to .3. Further, temperature meaurement for children and staff seems to be highly correlated in all waves, with correlations between .5 and .6. For the other variables, the correlations are usually between -.1 to .1

    – Hygiene masks includes both, staff wearing a face mask with other staff/parents as well as staff wearing a face mask with children (range 0 to 2).

    – Hygiene desinf. and vent. includes surface desinfection and regular ventilation (range 0 to 2).

    – Hygiene contact includes the three items on contact, group separation indoor and outdoor and fixed staff assignment to groups (range 0 to 3).

- Hygiene tests includes Covid-19 tests for children and staff as well as temperature measurement for children and staff (range 0 to 4).

- Appendix Figure A8 is a replication of Figure 4 showing coefficients from models that include all single hygiene variables at once, assuming different effects of every single measure here (generous setting). In the following, we briefly discuss findings from both settings and explain how they influenced our variable selection.

- Considering **face masks**, the summarized index in Figure A7 provides significant effects of face masks on the number of infections in children (wave 4a, 5) and for the occurrence and number of infections in staff (wave 4b, 5). Disentangeling the mask effect by using both variables in Figure A8, the use of face masks with staff/parents and with children shows that most of the protective effects stems from wearing face masks with children, while the usage of face masks in contact with staff/parents only showed significant negative effects for the number of infections in children in wave 4a. The protective effect on the occurrence of infections in staff in wave 5 found in Figure A7 fails to reach significance in Figure 4. Here, including the single items clearly provides helpful insights.

- When it comes to the index for **surface desinfection** and **regular ventilation** (Hygiene desinf. and vent), we cannot find any significant effects when using the index (see Figure A7). This supported our decision not to include surface desinfection in the final model. Including regular ventilation only (see Figure 4) or including both variables (see Figure A8) did not change that picture. Anyway, as regular ventilation was in focus of the debate in Germany, we decided to present the results in Figure 4, but to exclude surface disinfection, as preceding analyses [2] and actual results did not show any substantial results.

- For the **contact restriction** index, we found negative significant effects for the occurrence of infections in parents (wave 4b) and the number of infections in staff in the index setting (wave 4b, see Figure A7). When adding all (correlated) contact variables at once in the models (see Figure A8), we find a negative effect on the number of infections in children in wave 5 for outdoor group separation, but a positive effect for fixed staff assignment in the same model. In our opinion, this positive finding is best explained by the high correlation of the contact variables mentioned above, since it makes little sense in terms of content. We further find a single negative within effect of outdoor group separation on the occurrence of infections in parents in wave 4b. This

effect is also found in Figure A7. The significant effect (see Figure 4) of indoor group separation on the number of infections turns insignificant in Figure A8, but is still found in Figure A7. Overall, as the majority of all significant estimates for contact restriction variables in both our settings are clearly negative, hence, preventive, we consider group separation to be beneficial to some degree, without, however, being able to specify the exact area of effect. These findings in mind, we decided to use the indoor contact variable as a usefull proxy for contact restrictions in the final model, because it showed the highest correlation with the other two contact variables.

- Considering **tests**, the index setting (see Figure A7) provides significant effects for the number of infections in children (wave 5) and for the number of infections in staff (wave 4b and 5). Both effects make intuitive sense, as tests with children and tests with staff should reveal infections in children and staff.
  - Looking at both **COVID-19 test variables** as single items in the generous setting (see Figure A8) reveals a positive effect of testing staff on infections of staff, which makes perfect sense, but no further effects of staff testing. The positive effects of tests on the number of infections in children in wave 5 and on the number infections in staff in wave 4 found in the index setting fail to reach significance when separated into single items. The corresponding effects point in the same direction, but are not individually significant. The protective effect of the introduction of tests for children on occurrence and number of infections in parents in wave 4a and 4b is only found in Figure A8 and Figure 4, this supports the assumtion of increased attention by parents due to the tests they must perform with their children mentioned in the paper.
  - Considering **temperature measurement**, our in-depth analysis in Figure A8 provides negative significant within effects of the introduction of temperature measurement for children on the occurrence and number of infections in children in wave 4a, and on the number of infections in staff in wave 5. These results are counterintuitive to some degree, as a positive result in temperature measurement should actually lead to a positive test and thus have a positive effect size, as it would increase the number of infections found, similar to the positive effect of tests in the index setting (see Figure A7). We assume that this result is due to a very limited case number, but also due high correlation of the both temperature measurement variables (See Figure A2).

* Case Numbers: Figures A4 to A6 show histograms of the within variance of all time-varying variables per wave. As Figures A4 to A6 show, the estimates for temperature measurement are based on comparatively few observations with within variance, as could be seen in the extremely small tails of the corresponding histograms. In numbers: Only 220 of 4721 centres in wave 4a have any within variance unequal to 0 in temperature measurements for children in wave 4a, and of these 220 centres, only 44 observe any infections in children, while e.g. 1625 centres have a within variance unequal to 0 when it comes to face masks for children in wave 4a (own calculations).
* Correlation: Looking at the other non-significant effects for temperature measurement for staff in Figure A8, e.g. in the first model (occurrance wave 4a), the two temperature measurement variables seem to work in different directions and have comparatively large confidence bounds. The within effect for temperature measurement for staff is comparably large and positive, but not significant, while temperature measurement for children is negative. This finding might result from the high correlation (.5 to .6, see Figure A2) between the two variables. To follow up further, we run an additional model where we include only temperature measurement for children and drop the temperature measurement for staff variable (see Figure A9). Here, the effect for temperature measurement for children is still negative, but not significant.

Hence, when it comes to temperature measurement, we find it somewhat difficult to make any substantive statement here. Moreover, given the very low case numbers, the lack of within variation and high correlation between both measurements, prefer to put the result under great reserve and refrain from including the variables into the final model. Further research is needed here, especially in areas where temperature measurement has been applied more widely.

Figure A7: Index Setting: Replication of Figure 4 including all hygiene measures shown in Figure 3 as indices

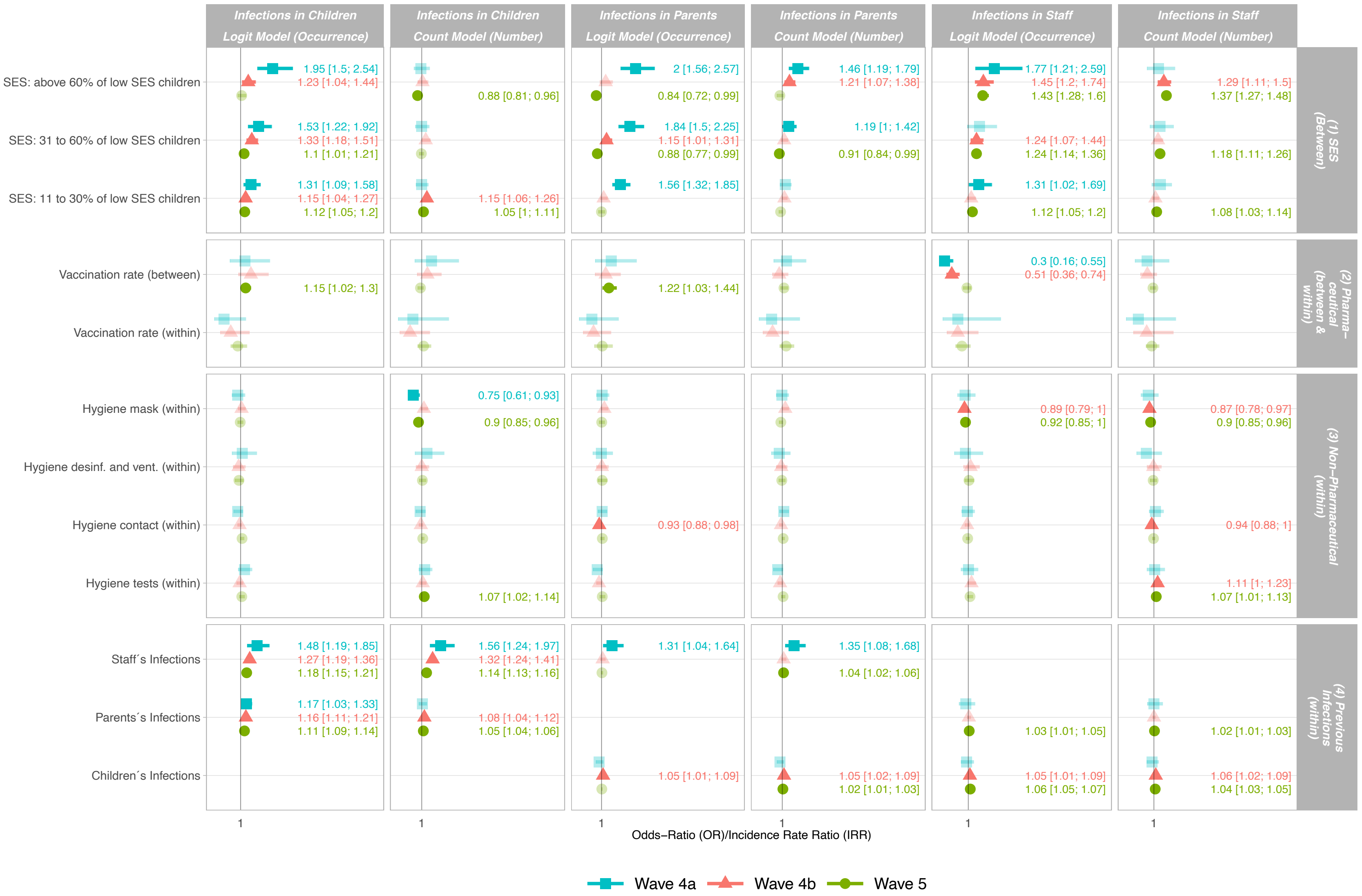

Source: ECEC centre registry, estimates from logit and count models for SARS-CoV-2 infections children, parents and staff. Odds ratios (OR, logit Models) and incidence rate ratios (IRR, count models), significant coefficients ($p < 0.05$) are printed in opaque with OR/IRR and 95% CI, non significant coefficients are printed in transparent colours. Full models in tables A6, A7 and A8, own calculations.

Figure A8: Generous setting: Replication of Figure 4 including all hygiene measures shown in Figure 3 as single items

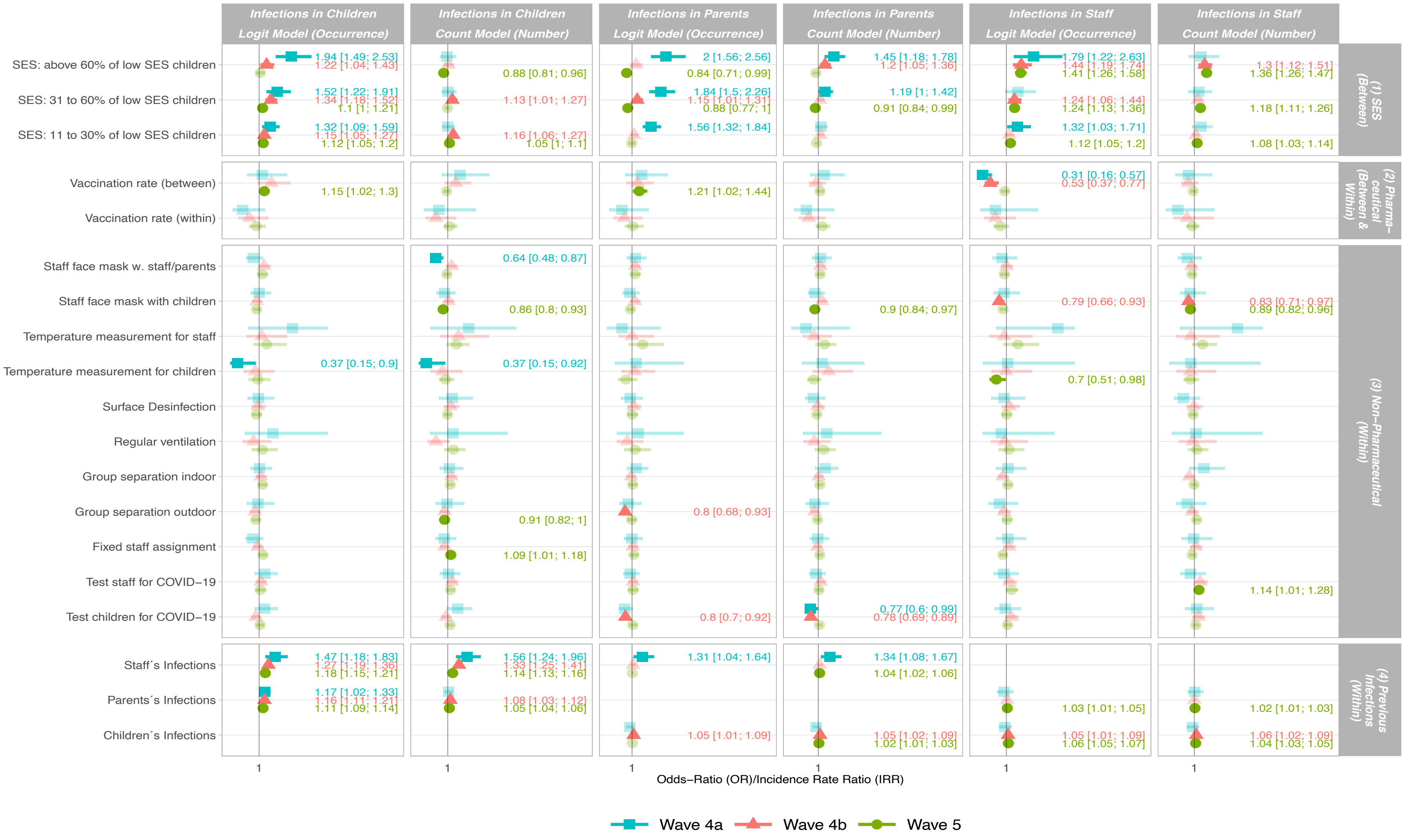

Source: ECEC centre registry, estimates from logit and count models for SARS-CoV-2 infections children, parents and staff. Odds ratios (OR, logit Models) and incidence rate ratios (IRR, count models), significant coefficients ($p < 0.05$) are printed in opaque with OR/IRR and 95% CI, non significant coefficients are printed in transparent colours. Full models in tables A9, A10 and A11, own calculations.

Figure A9: Generous setting: Replication of Figure 4 including all hygiene measures shown in Figure 3 as single items, includes only temperature measurement for children

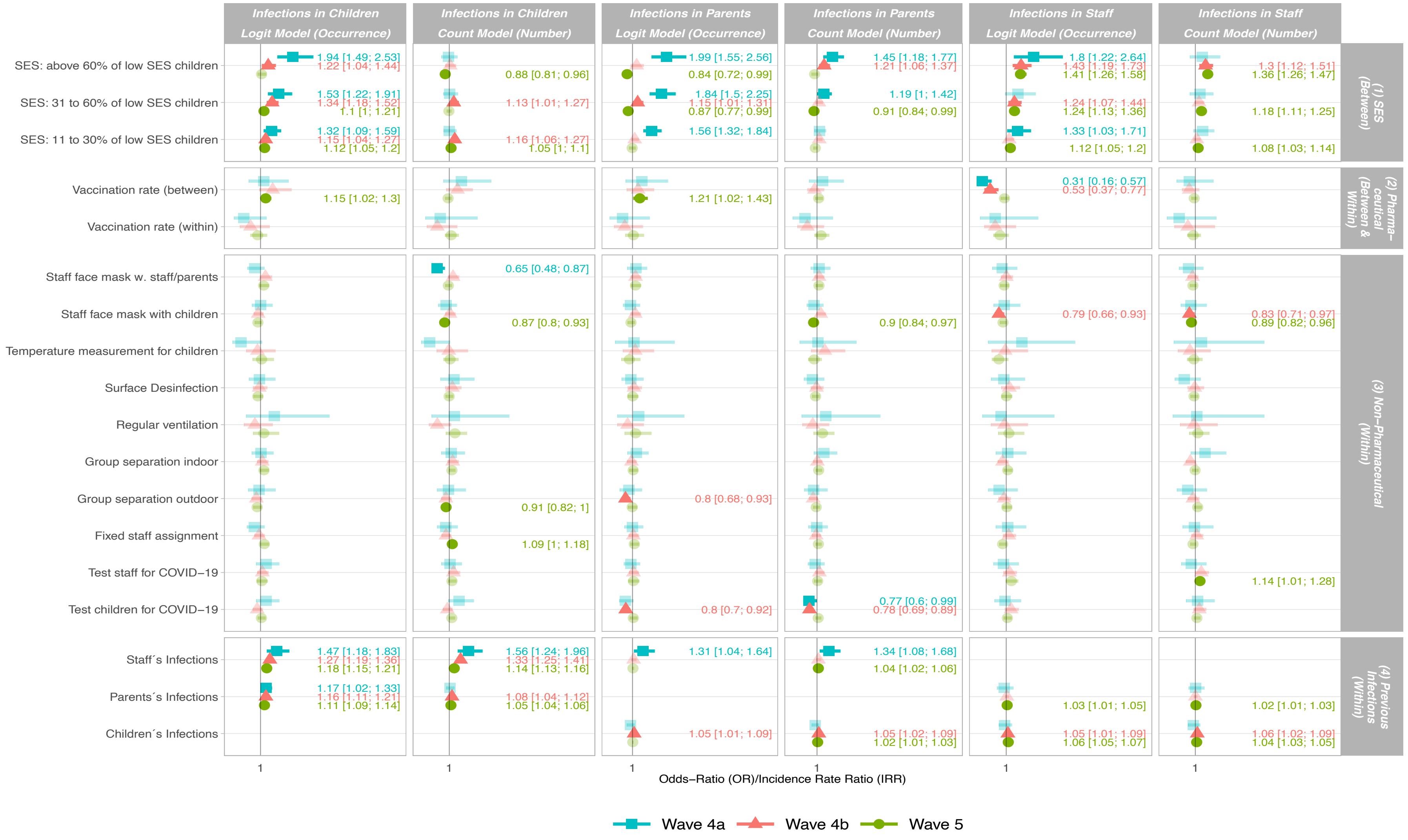

Source: ECEC centre registry, estimates from logit and count models for SARS-CoV-2 infections children, parents and staff. Odds ratios (OR, logit Models) and incidence rate ratios (IRR, count models), significant coefficients ($p < 0.05$) are printed in opaque with OR/IRR and 95% CI, non significant coefficients are printed in transparent colours. Full models not shown, own calculations.

Table A6: Logit and count models for infections in children, parents and staff, wave 4a

	(1): Child, 4a occurrence	(2): Child, 4a. number	(3): Parent, 4a. occurrence	(4): Parent, 4a. number	(5): Staff, 4a occurrence	(6): Staff, 4a number
Intercept	−5.58*	−2.68*	−5.34*	−2.38*	−4.78*	−1.86*
	[−6.30; −4.85]	[−3.45; −1.91]	[−6.06; −4.62]	[−3.06; −1.70]	[−5.66; −3.90]	[−2.83; −0.89]
SES: 11 to 30% of low SES children (ref.: below 10%)	0.27*	−0.01	0.45*	0.08	0.27*	0.18
	[0.08; 0.46]	[−0.19; 0.18]	[0.28; 0.61]	[−0.07; 0.24]	[0.02; 0.53]	[−0.06; 0.42]
SES: 31 to 60% of low SES children	0.42*	−0.01	0.61*	0.17*	0.29	0.17
	[0.20; 0.65]	[−0.21; 0.20]	[0.41; 0.81]	[0.00; 0.35]	[−0.03; 0.61]	[−0.13; 0.46]
SES: above 60% of low SES children	0.67*	−0.03	0.69*	0.38*	0.57*	0.13
	[0.40; 0.93]	[−0.26; 0.21]	[0.45; 0.94]	[0.18; 0.58]	[0.19; 0.95]	[−0.23; 0.48]
Hyg: Desinf. and vent. (between)	0.00	0.12	−0.03	0.01	−0.10	−0.23
	[−0.25; 0.26]	[−0.14; 0.38]	[−0.26; 0.20]	[−0.20; 0.22]	[−0.44; 0.25]	[−0.57; 0.11]
Hyg: Tests (between)	0.08	0.07	−0.02	0.02	0.06	−0.11
	[−0.03; 0.19]	[−0.04; 0.18]	[−0.12; 0.08]	[−0.07; 0.10]	[−0.09; 0.22]	[−0.27; 0.04]
Hyg: Contact (between)	0.02	0.02	0.02	−0.01	0.13*	0.01
	[−0.05; 0.10]	[−0.05; 0.09]	[−0.05; 0.09]	[−0.07; 0.05]	[0.03; 0.24]	[−0.09; 0.11]
Hyg: Mask (between)	0.14*	−0.00	0.18*	0.04	0.11	0.07
	[0.01; 0.28]	[−0.14; 0.13]	[0.06; 0.30]	[−0.07; 0.15]	[−0.08; 0.30]	[−0.13; 0.28]
Hyg: Desinf. and vent. (within)	0.05	0.14	−0.01	−0.09	−0.09	−0.26
	[−0.29; 0.39]	[−0.22; 0.51]	[−0.30; 0.28]	[−0.39; 0.21]	[−0.53; 0.36]	[−0.72; 0.21]
Hyg: Tests (within)	0.11	0.09	−0.13	−0.15	0.00	0.02
	[−0.08; 0.29]	[−0.09; 0.27]	[−0.29; 0.03]	[−0.31; 0.01]	[−0.25; 0.25]	[−0.23; 0.28]
Hyg: Contact (within)	−0.09	−0.05	0.02	0.04	−0.02	0.05
	[−0.23; 0.06]	[−0.20; 0.10]	[−0.11; 0.15]	[−0.09; 0.17]	[−0.22; 0.17]	[−0.15; 0.25]
Hyg: Mask (within)	−0.09	−0.28*	0.02	−0.01	−0.10	−0.18
	[−0.30; 0.12]	[−0.50; −0.07]	[−0.17; 0.20]	[−0.20; 0.17]	[−0.38; 0.18]	[−0.48; 0.12]
Vaccination rate (between)	0.12	0.25	0.25	0.12	−1.22*	−0.23
	[−0.38; 0.62]	[−0.23; 0.74]	[−0.21; 0.71]	[−0.29; 0.53]	[−1.83; −0.60]	[−0.82; 0.37]
Vaccination rate (within)	−0.68	−0.30	−0.34	−0.38	−0.37	−0.61
	[−1.50; 0.14]	[−1.20; 0.59]	[−1.09; 0.41]	[−1.18; 0.41]	[−1.41; 0.67]	[−1.72; 0.50]
Nr. of Infections: Staff (between)	2.77*	1.00*	0.34	0.05		
	[1.87; 3.67]	[0.36; 1.64]	[−0.61; 1.29]	[−0.59; 0.68]		
Nr. of Infections: Staff (within)	0.40*	0.45*	0.27*	0.30*		
	[0.18; 0.61]	[0.22; 0.68]	[0.04; 0.50]	[0.08; 0.52]		
Nr. of Infections: Parents (between)	3.11*	0.82*			−0.03	0.27
	[2.69; 3.53]	[0.59; 1.05]			[−0.68; 0.63]	[−0.19; 0.74]
Nr. of Infections: Parents (within)	0.16*	0.02			−0.07	0.01
	[0.03; 0.29]	[−0.09; 0.14]			[−0.32; 0.18]	[−0.22; 0.23]
7 day Incidence (within)	0.01*	0.01*	0.01*	0.01*	0.02*	0.01*
	[0.01; 0.02]	[0.01; 0.02]	[0.01; 0.01]	[0.01; 0.01]	[0.01; 0.02]	[0.00; 0.01]
7 day Incidence (between)	0.00*	−0.00	0.00*	−0.00	0.01	−0.00
	[0.00; 0.01]	[−0.01; 0.00]	[0.00; 0.01]	[−0.00; 0.00]	[−0.00; 0.01]	[−0.01; 0.01]
Increase 7 day Incidence (t to t+x, within)	0.00*	0.01*	0.00*	0.00*	0.00*	0.01*
	[0.00; 0.01]	[0.00; 0.01]	[0.00; 0.00]	[0.00; 0.01]	[0.00; 0.01]	[0.00; 0.01]
Increase 7 day Incidence (t to t+x, between)	0.00*	0.01*	0.00*	0.01*	0.00*	−0.00
	[0.00; 0.01]	[0.00; 0.01]	[0.00; 0.00]	[0.00; 0.01]	[0.00; 0.00]	[−0.01; 0.01]
Nr. of Infections: Children (between)			3.42*	1.20*	1.97*	0.70*
			[2.97; 3.87]	[0.93; 1.47]	[1.22; 2.72]	[0.20; 1.20]
Nr. of Infections: Children (within)			−0.07	−0.07	−0.05	−0.05
			[−0.18; 0.05]	[−0.17; 0.04]	[−0.25; 0.15]	[−0.24; 0.13]
AIC	7711.35	5817.27	9763.06	7913.78	4825.65	2788.01
Log Likelihood	−3825.67	−2877.64	−4851.53	−3925.89	−2382.82	−1363.00
Num. obs.	36472	5716	36472	7448	36472	3071
Num. groups: week	13	13	13	13	13	13
Num. groups: token	4721	655	4721	851	4721	352
Var: week (Intercept)	0.16	0.27	0.42	0.30	0.04	0.43
Var: token (Intercept)	0.96	0.00	1.05	0.00	1.79	0.00

$^{*}p < 0.05$, All estimates as log odds, own calculations.

Table A7: Logit and count models for infections in children, parents and staff, wave 4b

	(1): Child, 4b occurrence	(2): Child, 4b. number	(3): Parent, 4b. occurrence	(4): Parent, 4b. number	(5): Staff, 4b occurrence	(6): Staff, 4b number
Intercept	−3.69*	−2.27*	−3.48*	−2.05*	−3.37*	−2.11*
	[−4.16; −3.22]	[−2.70; −1.83]	[−3.97; −2.99]	[−2.44; −1.66]	[−3.90; −2.83]	[−2.58; −1.64]
SES: 11 to 30% of low SES children (ref.: below 10%)	0.14*	0.14*	0.06	0.06	0.08	0.04
	[0.04; 0.24]	[0.05; 0.23]	[−0.04; 0.16]	[−0.01; 0.14]	[−0.03; 0.20]	[−0.05; 0.14]
SES: 31 to 60% of low SES children	0.29*	0.11	0.14*	0.05	0.22*	0.12
	[0.16; 0.41]	[−0.00; 0.23]	[0.01; 0.27]	[−0.04; 0.15]	[0.07; 0.37]	[−0.00; 0.23]
SES: above 60% of low SES children	0.20*	0.02	0.11	0.19*	0.37*	0.26*
	[0.04; 0.37]	[−0.13; 0.17]	[−0.05; 0.28]	[0.07; 0.32]	[0.18; 0.56]	[0.11; 0.41]
Hyg: Desinf. and vent. (between)	−0.17*	0.03	0.05	0.05	−0.09	0.05
	[−0.30; −0.05]	[−0.08; 0.15]	[−0.08; 0.18]	[−0.05; 0.15]	[−0.23; 0.06]	[−0.07; 0.17]
Hyg: Tests (between)	0.13*	0.02	0.02	−0.03	0.01	0.06
	[0.07; 0.19]	[−0.03; 0.08]	[−0.04; 0.08]	[−0.07; 0.02]	[−0.06; 0.08]	[−0.00; 0.12]
Hyg: Contact (between)	0.03	0.04	0.01	0.02	0.04	−0.02
	[−0.01; 0.08]	[−0.00; 0.08]	[−0.04; 0.06]	[−0.01; 0.06]	[−0.01; 0.10]	[−0.06; 0.03]
Hyg: Mask (between)	0.09*	0.01	0.06	0.03	0.06	−0.00
	[0.01; 0.16]	[−0.05; 0.08]	[−0.01; 0.13]	[−0.02; 0.09]	[−0.02; 0.14]	[−0.07; 0.06]
Hyg: Desinf. and vent. (within)	−0.06	0.00	0.01	−0.03	0.07	−0.02
	[−0.25; 0.13]	[−0.19; 0.19]	[−0.17; 0.18]	[−0.20; 0.13]	[−0.15; 0.28]	[−0.23; 0.20]
Hyg: Tests (within)	−0.03	0.03	−0.08	−0.07	0.09	0.11*
	[−0.12; 0.07]	[−0.06; 0.12]	[−0.16; 0.01]	[−0.15; 0.01]	[−0.02; 0.20]	[0.00; 0.21]
Hyg: Contact (within)	−0.03	−0.03	−0.07*	−0.04	−0.04	−0.07*
	[−0.09; 0.03]	[−0.09; 0.03]	[−0.13; −0.02]	[−0.09; 0.01]	[−0.11; 0.03]	[−0.13; −0.00]
Hyg: Mask (within)	0.03	0.06	0.08	0.08	−0.12*	−0.14*
	[−0.07; 0.13]	[−0.03; 0.16]	[−0.01; 0.17]	[−0.00; 0.17]	[−0.24; −0.00]	[−0.25; −0.03]
Vaccination rate (between)	0.27	0.16	0.12	−0.10	−0.67*	−0.22
	[−0.07; 0.60]	[−0.15; 0.46]	[−0.21; 0.44]	[−0.35; 0.16]	[−1.04; −0.31]	[−0.51; 0.08]
Vaccination rate (within)	−0.35	−0.42	−0.27	−0.34	−0.37	−0.24
	[−0.92; 0.23]	[−1.05; 0.21]	[−0.78; 0.24]	[−0.85; 0.17]	[−1.00; 0.26]	[−0.93; 0.45]
Nr. of Infections: Staff (between)	1.18*	1.01*	0.04	0.17		
	[1.00; 1.36]	[0.87; 1.15]	[−0.18; 0.26]	[−0.00; 0.34]		
Nr. of Infections: Staff (within)	0.24*	0.28*	0.03	0.04		
	[0.17; 0.30]	[0.22; 0.34]	[−0.04; 0.10]	[−0.03; 0.10]		
Nr. of Infections: Parents (between)	1.26*	0.64*			0.34*	0.09
	[1.14; 1.37]	[0.55; 0.73]			[0.20; 0.48]	[−0.01; 0.19]
Nr. of Infections: Parents (within)	0.14*	0.07*			0.02	0.00
	[0.10; 0.19]	[0.03; 0.12]			[−0.03; 0.07]	[−0.04; 0.05]
7 day Incidence (within)	0.00*	0.00*	0.00*	0.00*	0.00*	0.00*
	[0.00; 0.00]	[0.00; 0.00]	[0.00; 0.00]	[0.00; 0.00]	[0.00; 0.00]	[0.00; 0.00]
7 day Incidence (between)	0.00	−0.00	0.00*	0.00*	0.00*	0.00
	[−0.00; 0.00]	[−0.00; 0.00]	[0.00; 0.00]	[0.00; 0.00]	[0.00; 0.00]	[−0.00; 0.00]
Increase 7 day Incidence (t to t+x, within)	0.00*	0.00*	0.00*	0.00*	0.00*	0.00*
	[0.00; 0.00]	[0.00; 0.00]	[0.00; 0.00]	[0.00; 0.00]	[0.00; 0.00]	[0.00; 0.00]
Increase 7 day Incidence (t to t+x, between)	0.00*	0.00*	0.00*	0.00*	0.00*	0.00*
	[0.00; 0.00]	[0.00; 0.00]	[0.00; 0.00]	[0.00; 0.00]	[0.00; 0.00]	[0.00; 0.00]
Nr. of Infections: Children (between)			0.78*	0.70*	0.83*	0.58*
			[0.66; 0.90]	[0.60; 0.79]	[0.71; 0.96]	[0.50; 0.66]
Nr. of Infections: Children (within)			0.04*	0.05*	0.05*	0.05*
			[0.01; 0.08]	[0.02; 0.08]	[0.01; 0.09]	[0.02; 0.09]
AIC	23390.82	27470.55	28494.46	34978.30	19362.84	18538.30
Log Likelihood	−11665.41	−13704.27	−14217.23	−17458.15	−9651.42	−9238.15
Num. obs.	40133	20183	40133	23826	40133	15756
Num. groups: week	13	13	13	13	13	13
Num. groups: token	4283	1999	4283	2326	4283	1558
Var: week (Intercept)	0.15	0.12	0.21	0.14	0.21	0.24
Var: token (Intercept)	0.44	0.10	0.70	0.09	0.68	0.02

$^{*}p < 0.05$, All estimates as log odds, own calculations.

Table A8: Logit and count models for infections in children, parents and staff, wave 5

	(1): Child, 5 occurrence	(2): Child, 5. number	(3): Parent, 5. occurrence	(4): Parent, 5. number	(5): Staff, 5 occurrence	(6): Staff, 5 number
Intercept	−2.22*	−0.85*	−1.58*	−0.91*	−1.86*	−1.22*
	[−2.52; −1.93]	[−1.08; −0.62]	[−1.97; −1.20]	[−1.16; −0.67]	[−2.15; −1.56]	[−1.44; −1.00]
SES: 11 to 30% of low SES children (ref.: below 10%)	0.12*	0.05*	−0.00	−0.05	0.12*	0.08*
	[0.05; 0.18]	[0.00; 0.10]	[−0.10; 0.09]	[−0.11; 0.01]	[0.05; 0.18]	[0.03; 0.13]
SES: 31 to 60% of low SES children	0.10*	−0.01	−0.13*	−0.09*	0.22*	0.17*
	[0.01; 0.19]	[−0.08; 0.06]	[−0.26; −0.01]	[−0.17; −0.01]	[0.13; 0.31]	[0.10; 0.23]
SES: above 60% of low SES children	0.03	−0.13*	−0.17*	−0.07	0.36*	0.32*
	[−0.09; 0.15]	[−0.22; −0.05]	[−0.33; −0.01]	[−0.17; 0.03]	[0.24; 0.47]	[0.24; 0.39]
Hyg: Desinf. and vent. (between)	0.07	0.03	0.14*	0.09*	−0.03	0.05
	[−0.02; 0.16]	[−0.04; 0.09]	[0.01; 0.27]	[0.02; 0.17]	[−0.11; 0.06]	[−0.02; 0.11]
Hyg: Tests (between)	0.11*	0.05*	0.08*	0.03	0.05	−0.02
	[0.05; 0.16]	[0.01; 0.08]	[0.00; 0.15]	[−0.01; 0.08]	[−0.01; 0.10]	[−0.06; 0.02]
Hyg: Contact (between)	0.02	0.01	−0.01	−0.00	−0.01	−0.03*
	[−0.00; 0.05]	[−0.01; 0.03]	[−0.05; 0.03]	[−0.03; 0.02]	[−0.03; 0.02]	[−0.05; −0.01]
Hyg: Mask (between)	0.06*	0.04*	0.08*	0.07*	0.03	−0.03
	[0.01; 0.12]	[0.00; 0.08]	[0.01; 0.15]	[0.03; 0.11]	[−0.02; 0.08]	[−0.07; 0.00]
Hyg: Desinf. and vent. (within)	−0.05	0.02	0.02	−0.01	0.02	−0.02
	[−0.18; 0.08]	[−0.08; 0.12]	[−0.12; 0.15]	[−0.11; 0.08]	[−0.11; 0.15]	[−0.12; 0.08]
Hyg: Tests (within)	0.03	0.07*	0.02	0.02	0.05	0.07*
	[−0.04; 0.11]	[0.02; 0.13]	[−0.05; 0.10]	[−0.03; 0.08]	[−0.03; 0.13]	[0.01; 0.13]
Hyg: Contact (within)	0.04	0.03	0.02	0.03	−0.02	−0.01
	[−0.01; 0.09]	[−0.01; 0.07]	[−0.03; 0.08]	[−0.01; 0.06]	[−0.07; 0.03]	[−0.05; 0.03]
Hyg: Mask (within)	−0.01	−0.10*	0.00	−0.04	−0.09*	−0.10*
	[−0.09; 0.07]	[−0.16; −0.04]	[−0.08; 0.09]	[−0.10; 0.01]	[−0.17; −0.00]	[−0.16; −0.04]
Vaccination rate (between)	0.14*	−0.04	0.20*	0.05	−0.04	−0.03
	[0.02; 0.26]	[−0.13; 0.05]	[0.03; 0.37]	[−0.06; 0.15]	[−0.16; 0.08]	[−0.11; 0.06]
Vaccination rate (within)	−0.09	0.05	0.02	0.11	−0.21	−0.06
	[−0.35; 0.17]	[−0.13; 0.24]	[−0.24; 0.28]	[−0.08; 0.29]	[−0.47; 0.06]	[−0.27; 0.14]
Nr. of Infections: Staff (between)	0.62*	0.59*	0.13*	0.15*		
	[0.55; 0.68]	[0.54; 0.63]	[0.04; 0.22]	[0.09; 0.20]		
Nr. of Infections: Staff (within)	0.17*	0.13*	0.01	0.04*		
	[0.14; 0.19]	[0.12; 0.15]	[−0.01; 0.04]	[0.02; 0.05]		
Nr. of Infections: Parents (between)	0.37*	0.18*			0.13*	0.04*
	[0.33; 0.40]	[0.16; 0.21]			[0.10; 0.16]	[0.02; 0.06]
Nr. of Infections: Parents (within)	0.11*	0.05*			0.03*	0.02*
	[0.09; 0.13]	[0.04; 0.06]			[0.01; 0.04]	[0.01; 0.03]
7 day Incidence (within)	0.00*	0.00*	0.00*	0.00*	0.00*	0.00*
	[0.00; 0.00]	[0.00; 0.00]	[0.00; 0.00]	[0.00; 0.00]	[0.00; 0.00]	[0.00; 0.00]
7 day Incidence (between)	0.00*	−0.00	0.00	−0.00	0.00*	0.00
	[0.00; 0.00]	[−0.00; 0.00]	[−0.00; 0.00]	[−0.00; 0.00]	[0.00; 0.00]	[−0.00; 0.00]
Increase 7 day Incidence (t to t+x, within)	0.00*	0.00*	0.00*	0.00*	0.00*	0.00*
	[0.00; 0.00]	[0.00; 0.00]	[0.00; 0.00]	[0.00; 0.00]	[0.00; 0.00]	[0.00; 0.00]
Increase 7 day Incidence (t to t+x, between)	0.00*	0.00*	−0.00	0.00*	0.00*	0.00*
	[0.00; 0.00]	[0.00; 0.00]	[−0.00; 0.00]	[0.00; 0.00]	[0.00; 0.00]	[0.00; 0.00]
Nr. of Infections: Children (between)			0.16*	0.21*	0.24*	0.23*
			[0.12; 0.20]	[0.18; 0.23]	[0.21; 0.27]	[0.22; 0.25]
Nr. of Infections: Children (within)			0.01	0.02*	0.06*	0.04*
			[−0.00; 0.02]	[0.01; 0.03]	[0.05; 0.07]	[0.03; 0.05]
AIC	38714.79	94838.41	39360.53	82133.98	38017.06	68258.99
Log Likelihood	−19327.40	−47388.20	−19650.26	−41035.99	−18978.53	−34098.49
Num. obs.	31030	30037	31030	28498	31030	29430
Num. groups: week	11	11	11	11	11	11
Num. groups: token	4073	3821	4073	3566	4073	3683
Var: week (Intercept)	0.03	0.04	0.03	0.02	0.05	0.04
Var: token (Intercept)	0.24	0.12	0.96	0.24	0.19	0.04

$^*p < 0.05$, All estimates as log odds, own calculations.

Table A9: Replication of Table A1 including all hygiene measures: Logit and count models for infections in children, parents and staff, wave 4a

	(1): Child, 4a occurrence	(2): Child, 4a. number	(3): Parent, 4a. occurrence	(4): Parent, 4a. number	(5): Staff, 4a occurrence	(6): Staff, 4a number
Intercept	−4.64*	−1.29	−4.85*	−2.97*	−4.00*	−1.11
	[−6.11; −3.17]	[−3.19; 0.61]	[−6.28; −3.42]	[−4.43; −1.51]	[−5.90; −2.10]	[−3.32; 1.09]
SES: 11 to 30% of low SES children (ref.: below 10%)	0.27*	0.00	0.44*	0.07	0.28*	0.18
	[0.09; 0.46]	[−0.18; 0.18]	[0.27; 0.61]	[−0.08; 0.23]	[0.02; 0.54]	[−0.07; 0.43]
SES: 31 to 60% of low SES children	0.42*	0.01	0.61*	0.18*	0.29	0.12
	[0.19; 0.65]	[−0.19; 0.22]	[0.41; 0.81]	[0.00; 0.35]	[−0.03; 0.62]	[−0.18; 0.41]
SES: above 60% of low SES children	0.66*	−0.03	0.69*	0.37*	0.58*	0.18
	[0.40; 0.93]	[−0.26; 0.21]	[0.44; 0.94]	[0.17; 0.58]	[0.20; 0.97]	[−0.18; 0.54]
Regular ventilation of rooms (between)	−1.00	−1.40	−0.54	0.57	−0.92	−0.99
	[−2.40; 0.40]	[−3.26; 0.45]	[−1.87; 0.79]	[−0.79; 1.92]	[−2.76; 0.93]	[−3.10; 1.12]
Regular ventilation of rooms (within)	0.34	0.14	0.17	0.23	−0.16	0.04
	[−0.55; 1.22]	[−0.73; 1.01]	[−0.59; 0.92]	[−0.59; 1.04]	[−1.19; 0.88]	[−1.07; 1.15]
Fixed staff assignment to groups (between)	−0.02	−0.06	−0.08	−0.07	0.27	0.55*
	[−0.29; 0.24]	[−0.32; 0.20]	[−0.32; 0.15]	[−0.29; 0.14]	[−0.09; 0.64]	[0.21; 0.89]
Fixed staff assignment to groups (within)	−0.20	−0.11	−0.00	−0.01	0.05	−0.02
	[−0.51; 0.11]	[−0.44; 0.22]	[−0.27; 0.27]	[−0.29; 0.27]	[−0.36; 0.45]	[−0.46; 0.42]
Group separation indoors (between)	0.22	0.17	0.12	−0.10	−0.06	−0.61*
	[−0.07; 0.50]	[−0.12; 0.45]	[−0.14; 0.38]	[−0.34; 0.14]	[−0.46; 0.35]	[−1.04; −0.17]
Group separation indoors (within)	0.01	0.05	0.11	0.18	0.04	0.24
	[−0.30; 0.31]	[−0.27; 0.37]	[−0.16; 0.38]	[−0.10; 0.46]	[−0.36; 0.45]	[−0.15; 0.64]
Group separation outdoors (between)	−0.19	−0.09	0.02	0.18	0.21	0.12
	[−0.47; 0.09]	[−0.35; 0.17]	[−0.23; 0.27]	[−0.04; 0.40]	[−0.17; 0.58]	[−0.27; 0.52]
Group separation outdoors (within)	−0.04	−0.02	−0.11	−0.10	−0.22	−0.23
	[−0.44; 0.36]	[−0.44; 0.39]	[−0.46; 0.24]	[−0.46; 0.27]	[−0.74; 0.29]	[−0.76; 0.29]
Regular surface disinfection (between)	0.06	0.16	−0.01	−0.03	−0.04	−0.14
	[−0.22; 0.33]	[−0.12; 0.44]	[−0.25; 0.23]	[−0.25; 0.20]	[−0.41; 0.33]	[−0.51; 0.23]
Regular surface disinfection (within)	−0.03	0.13	−0.06	−0.15	−0.07	−0.38
	[−0.41; 0.36]	[−0.30; 0.55]	[−0.38; 0.27]	[−0.48; 0.18]	[−0.58; 0.43]	[−0.91; 0.14]
Temperature measurement for staff (between)	−0.03	0.25	0.65*	−0.25	0.04	0.26
	[−0.74; 0.68]	[−0.46; 0.96]	[0.03; 1.27]	[−0.79; 0.28]	[−0.98; 1.06]	[−0.88; 1.40]
Temperature measurement for staff (within)	0.68	0.48	−0.35	−0.45	0.92	0.82
	[−0.36; 1.72]	[−0.71; 1.67]	[−1.30; 0.60]	[−1.55; 0.65]	[−0.35; 2.19]	[−0.55; 2.19]
Temperature measurement for children (between)	0.04	−0.01	−0.32	0.16	−0.03	−0.24
	[−0.42; 0.50]	[−0.47; 0.46]	[−0.76; 0.12]	[−0.24; 0.57]	[−0.68; 0.62]	[−1.02; 0.53]
Temperature measurement for children (within)	−1.00*	−0.98*	0.12	0.11	0.04	−0.11
	[−1.89; −0.11]	[−1.88; −0.08]	[−0.69; 0.92]	[−0.64; 0.85]	[−1.16; 1.23]	[−1.30; 1.07]
Staff face mask with staff/parents (between)	0.23	−0.06	0.26*	0.05	−0.03	−0.10
	[−0.01; 0.48]	[−0.31; 0.18]	[0.05; 0.48]	[−0.15; 0.25]	[−0.36; 0.30]	[−0.44; 0.23]
Staff face mask with staff/parents (within)	−0.19	−0.44*	0.09	0.06	−0.13	−0.23
	[−0.48; 0.10]	[−0.73; −0.14]	[−0.17; 0.35]	[−0.20; 0.32]	[−0.52; 0.27]	[−0.66; 0.19]
Staff face mask with children (between)	0.10	0.02	0.13	0.03	0.22	0.23
	[−0.10; 0.30]	[−0.17; 0.22]	[−0.05; 0.30]	[−0.13; 0.19]	[−0.05; 0.49]	[−0.05; 0.51]
Staff face mask with children (within)	0.00	−0.10	−0.06	−0.10	−0.07	−0.13
	[−0.29; 0.29]	[−0.39; 0.19]	[−0.32; 0.19]	[−0.36; 0.16]	[−0.47; 0.33]	[−0.55; 0.28]
Test staff for COVID-19 (between)	0.03	0.17	−0.06	0.11	0.06	−0.20
	[−0.22; 0.28]	[−0.07; 0.41]	[−0.28; 0.15]	[−0.09; 0.30]	[−0.29; 0.41]	[−0.53; 0.13]
Test staff for COVID-19 (within)	0.15	0.02	−0.05	−0.06	−0.08	−0.13
	[−0.14; 0.43]	[−0.27; 0.31]	[−0.29; 0.19]	[−0.31; 0.19]	[−0.47; 0.31]	[−0.53; 0.28]
Test children for COVID-19 (between)	0.13	−0.00	−0.03	−0.05	0.10	−0.09
	[−0.06; 0.33]	[−0.19; 0.19]	[−0.21; 0.15]	[−0.20; 0.11]	[−0.17; 0.37]	[−0.36; 0.18]
Test children for COVID-19 (within)	0.15	0.25	−0.23	−0.26*	−0.04	0.07
	[−0.14; 0.43]	[−0.03; 0.53]	[−0.49; 0.02]	[−0.51; −0.01]	[−0.42; 0.35]	[−0.31; 0.45]
Vaccination rate (between)	0.08	0.30	0.25	0.15	−1.19*	−0.20
	[−0.42; 0.59]	[−0.19; 0.80]	[−0.22; 0.71]	[−0.27; 0.57]	[−1.81; −0.57]	[−0.80; 0.41]
Vaccination rate (within)	−0.66	−0.30	−0.35	−0.42	−0.39	−0.66
	[−1.48; 0.16]	[−1.19; 0.60]	[−1.10; 0.40]	[−1.22; 0.38]	[−1.43; 0.65]	[−1.78; 0.46]
Nr. of Infections: Staff (between)	2.79*	1.04*	0.38	0.03		
	[1.88; 3.70]	[0.40; 1.68]	[−0.58; 1.33]	[−0.61; 0.67]		
Nr. of Infections: Staff (within)	0.39*	0.44*	0.27*	0.29*		
	[0.17; 0.60]	[0.22; 0.67]	[0.04; 0.49]	[0.07; 0.52]		
Nr. of Infections: Parents (between)	3.10*	0.82*			−0.02	0.27
	[2.68; 3.52]	[0.59; 1.05]			[−0.68; 0.64]	[−0.19; 0.74]
Nr. of Infections: Parents (within)	0.15*	0.02			−0.06	0.01
	[0.02; 0.28]	[−0.09; 0.14]			[−0.31; 0.19]	[−0.22; 0.23]
7 day Incidence (within)	0.01*	0.01*	0.01*	0.01*	0.02*	0.01*
	[0.01; 0.02]	[0.01; 0.02]	[0.01; 0.01]	[0.01; 0.01]	[0.01; 0.02]	[0.00; 0.01]
7 day Incidence (between)	0.00*	−0.00	0.00*	−0.00	0.01	−0.00
	[0.00; 0.01]	[−0.01; 0.00]	[0.00; 0.01]	[−0.00; 0.00]	[−0.00; 0.01]	[−0.01; 0.00]
Increase 7 day Incidence (t to t+x, within)	0.00*	0.01*	0.00*	0.00*	0.00*	0.01*
	[0.00; 0.01]	[0.00; 0.01]	[0.00; 0.00]	[0.00; 0.01]	[0.00; 0.01]	[0.00; 0.01]
Increase 7 day Incidence (t to t+x, between)	0.00*	0.01*	0.00*	0.01*	0.00*	−0.00
	[0.00; 0.01]	[0.00; 0.01]	[0.00; 0.00]	[0.00; 0.01]	[0.00; 0.00]	[−0.01; 0.01]
Nr. of Infections: Children (between)			3.40*	1.20*	1.97*	0.75*
			[2.95; 3.85]	[0.93; 1.48]	[1.22; 2.73]	[0.24; 1.26]
Nr. of Infections: Children (within)			−0.07	−0.06	−0.05	−0.06
			[−0.18; 0.05]	[−0.17; 0.05]	[−0.25; 0.15]	[−0.24; 0.12]
AIC	7733.01	5837.76	9792.28	7944.79	4847.76	2797.74
Log Likelihood	−3822.51	−2873.88	−4852.14	−3927.39	−2379.88	−1353.87
Num. obs.	36472	5716	36472	7448	36472	3071
Num. groups: week	13	13	13	13	13	13
Num. groups: token	4721	655	4721	851	4721	352
Var: week (Intercept)	0.16	0.27	0.43	0.32	0.03	0.41
Var: token (Intercept)	0.97	0.00	1.06	0.00	1.82	0.00

$^*p < 0.05$, All estimates as log odds, own calculations.

Table A10: Replication of Table A2 including all hygiene measures: Logit and count models for infections in children, parents and staff, wave 4b

	(1): Child, 4b occurrence	(2): Child, 4b. number	(3): Parent, 4b. occurrence	(4): Parent, 4b. number	(5): Staff, 4b occurrence	(6): Staff, 4b number
Intercept	−3.98*	−2.75*	−3.23*	−2.15*	−2.58*	−1.95*
	[−4.83; −3.13]	[−3.62; −1.88]	[−4.07; −2.39]	[−2.81; −1.49]	[−3.45; −1.71]	[−2.67; −1.23]
SES: 11 to 30% of low SES children (ref.: below 10%)	0.14*	0.15*	0.06	0.06	0.08	0.04
	[0.04; 0.24]	[0.06; 0.24]	[−0.04; 0.16]	[−0.01; 0.14]	[−0.03; 0.20]	[−0.05; 0.13]
SES: 31 to 60% of low SES children	0.29*	0.13*	0.14*	0.05	0.21*	0.10
	[0.17; 0.42]	[0.01; 0.24]	[0.01; 0.27]	[−0.04; 0.15]	[0.06; 0.36]	[−0.02; 0.22]
SES: above 60% of low SES children	0.20*	0.02	0.11	0.18*	0.36*	0.26*
	[0.04; 0.36]	[−0.13; 0.16]	[−0.06; 0.28]	[0.05; 0.31]	[0.18; 0.55]	[0.11; 0.41]
Regular ventilation of rooms (between)	0.02	0.43	−0.29	0.13	−1.08*	−0.18
	[−0.74; 0.78]	[−0.37; 1.23]	[−1.03; 0.45]	[−0.44; 0.71]	[−1.83; −0.32]	[−0.79; 0.43]
Regular ventilation of rooms (within)	−0.18	−0.42	−0.15	−0.13	−0.05	−0.05
	[−0.66; 0.30]	[−0.89; 0.05]	[−0.58; 0.28]	[−0.56; 0.30]	[−0.59; 0.48]	[−0.59; 0.50]
Fixed staff assignment to groups (between)	−0.02	−0.01	0.07	0.09	0.07	0.03
	[−0.16; 0.13]	[−0.14; 0.12]	[−0.07; 0.22]	[−0.02; 0.20]	[−0.10; 0.24]	[−0.11; 0.18]
Fixed staff assignment to groups (within)	−0.04	−0.10	0.02	−0.03	0.07	0.04
	[−0.20; 0.11]	[−0.25; 0.05]	[−0.12; 0.16]	[−0.16; 0.10]	[−0.10; 0.24]	[−0.12; 0.20]
Group separation indoors (between)	0.19*	0.15*	−0.06	−0.07	0.09	−0.11
	[0.04; 0.35]	[0.01; 0.30]	[−0.22; 0.10]	[−0.20; 0.05]	[−0.10; 0.28]	[−0.27; 0.05]
Group separation indoors (within)	0.05	0.09	−0.03	0.01	−0.11	−0.16
	[−0.10; 0.20]	[−0.05; 0.24]	[−0.17; 0.11]	[−0.11; 0.14]	[−0.29; 0.06]	[−0.32; 0.01]
Group separation outdoors (between)	−0.15	−0.06	0.02	0.07	−0.07	0.03
	[−0.32; 0.03]	[−0.22; 0.09]	[−0.15; 0.20]	[−0.07; 0.20]	[−0.28; 0.13]	[−0.14; 0.20]
Group separation outdoors (within)	−0.13	−0.10	−0.23*	−0.12	−0.07	−0.07
	[−0.30; 0.04]	[−0.26; 0.06]	[−0.38; −0.07]	[−0.26; 0.02]	[−0.26; 0.12]	[−0.25; 0.11]
Regular surface disinfection (between)	−0.19*	0.02	0.06	0.05	−0.02	0.07
	[−0.32; −0.06]	[−0.10; 0.14]	[−0.07; 0.20]	[−0.06; 0.15]	[−0.18; 0.14]	[−0.06; 0.20]
Regular surface disinfection (within)	−0.04	0.08	0.04	−0.02	0.09	−0.02
	[−0.25; 0.17]	[−0.13; 0.29]	[−0.15; 0.24]	[−0.20; 0.17]	[−0.16; 0.33]	[−0.25; 0.22]
Temperature measurement for staff (between)	0.32	0.46*	0.26	0.33*	−0.39	−0.15
	[−0.07; 0.72]	[0.10; 0.82]	[−0.14; 0.66]	[0.03; 0.64]	[−0.90; 0.13]	[−0.63; 0.33]
Temperature measurement for staff (within)	0.07	0.27	−0.00	−0.14	−0.07	−0.11
	[−0.45; 0.60]	[−0.25; 0.79]	[−0.49; 0.49]	[−0.60; 0.33]	[−0.70; 0.56]	[−0.77; 0.55]
Temperature measurement for children (between)	−0.04	−0.31*	−0.12	−0.17	0.02	−0.00
	[−0.30; 0.23]	[−0.57; −0.06]	[−0.39; 0.14]	[−0.38; 0.04]	[−0.29; 0.33]	[−0.28; 0.28]
Temperature measurement for children (within)	−0.12	−0.17	0.08	0.26	−0.01	−0.12
	[−0.60; 0.37]	[−0.68; 0.35]	[−0.36; 0.51]	[−0.17; 0.70]	[−0.57; 0.56]	[−0.73; 0.49]
Staff face mask with staff/parents (between)	0.12*	−0.03	0.08	0.02	0.15*	0.03
	[0.00; 0.24]	[−0.15; 0.08]	[−0.04; 0.20]	[−0.07; 0.11]	[0.01; 0.29]	[−0.08; 0.15]
Staff face mask with staff/parents (within)	0.13	0.11	0.08	0.06	0.00	−0.09
	[−0.02; 0.28]	[−0.04; 0.25]	[−0.05; 0.22]	[−0.07; 0.18]	[−0.17; 0.17]	[−0.25; 0.07]
Staff face mask with children (between)	0.05	0.04	0.02	0.03	−0.01	−0.02
	[−0.06; 0.15]	[−0.06; 0.14]	[−0.08; 0.13]	[−0.05; 0.12]	[−0.14; 0.12]	[−0.13; 0.09]
Staff face mask with children (within)	−0.07	0.02	0.08	0.11	−0.24*	−0.19*
	[−0.22; 0.08]	[−0.12; 0.16]	[−0.06; 0.21]	[−0.02; 0.23]	[−0.41; −0.07]	[−0.35; −0.03]
Test staff for COVID-19 (between)	0.25*	0.16*	0.11	0.01	0.13	0.09
	[0.11; 0.40]	[0.03; 0.30]	[−0.03; 0.25]	[−0.10; 0.12]	[−0.04; 0.29]	[−0.05; 0.24]
Test staff for COVID-19 (within)	0.05	0.11	0.03	0.07	0.09	0.16
	[−0.10; 0.20]	[−0.04; 0.26]	[−0.11; 0.16]	[−0.06; 0.19]	[−0.09; 0.26]	[−0.01; 0.33]
Test children for COVID-19 (between)	0.08	−0.02	−0.03	−0.07	−0.01	0.08
	[−0.02; 0.18]	[−0.11; 0.07]	[−0.13; 0.07]	[−0.15; 0.00]	[−0.13; 0.10]	[−0.02; 0.17]
Test children for COVID-19 (within)	−0.10	−0.06	−0.22*	−0.24*	0.13	0.11
	[−0.26; 0.05]	[−0.20; 0.09]	[−0.36; −0.08]	[−0.37; −0.12]	[−0.04; 0.31]	[−0.05; 0.27]
Vaccination rate (between)	0.31	0.21	0.15	−0.07	−0.63*	−0.19
	[−0.03; 0.64]	[−0.09; 0.52]	[−0.18; 0.49]	[−0.32; 0.19]	[−1.00; −0.27]	[−0.49; 0.10]
Vaccination rate (within)	−0.34	−0.42	−0.25	−0.33	−0.39	−0.25
	[−0.92; 0.23]	[−1.05; 0.21]	[−0.77; 0.26]	[−0.84; 0.18]	[−1.02; 0.24]	[−0.94; 0.44]
Nr. of Infections: Staff (between)	1.17*	1.01*	0.04	0.18*		
	[0.99; 1.35]	[0.87; 1.15]	[−0.18; 0.26]	[0.01; 0.36]		
Nr. of Infections: Staff (within)	0.24*	0.28*	0.03	0.04		
	[0.17; 0.31]	[0.22; 0.35]	[−0.04; 0.10]	[−0.03; 0.10]		
Nr. of Infections: Parents (between)	1.25*	0.63*			0.35*	0.09
	[1.14; 1.37]	[0.54; 0.72]			[0.21; 0.49]	[−0.01; 0.19]
Nr. of Infections: Parents (within)	0.14*	0.07*			0.02	0.00
	[0.10; 0.19]	[0.03; 0.12]			[−0.03; 0.07]	[−0.04; 0.05]
7 day Incidence (within)	0.00*	0.00*	0.00*	0.00*	0.00*	0.00*
	[0.00; 0.00]	[0.00; 0.00]	[0.00; 0.00]	[0.00; 0.00]	[0.00; 0.00]	[0.00; 0.00]
7 day Incidence (between)	0.00	−0.00	0.00*	0.00*	0.00*	0.00
	[−0.00; 0.00]	[−0.00; 0.00]	[0.00; 0.00]	[0.00; 0.00]	[0.00; 0.00]	[−0.00; 0.00]
Increase 7 day Incidence (t to t+x, within)	0.00*	0.00*	0.00*	0.00*	0.00*	0.00*
	[0.00; 0.00]	[0.00; 0.00]	[0.00; 0.00]	[0.00; 0.00]	[0.00; 0.00]	[0.00; 0.00]
Increase 7 day Incidence (t to t+x, between)	0.00*	0.00*	0.00*	0.00*	0.00*	0.00*
	[0.00; 0.00]	[0.00; 0.01]	[0.00; 0.00]	[0.00; 0.00]	[0.00; 0.00]	[0.00; 0.00]
Nr. of Infections: Children (between)			0.78*	0.70*	0.83*	0.59*
			[0.66; 0.90]	[0.60; 0.79]	[0.71; 0.96]	[0.51; 0.67]
Nr. of Infections: Children (within)			0.05*	0.05*	0.05*	0.05*
			[0.01; 0.08]	[0.02; 0.09]	[0.01; 0.09]	[0.02; 0.09]
AIC	23425.48	27497.54	28531.57	35014.11	19389.66	18581.33
Log Likelihood	−11668.74	−13703.77	−14221.78	−17462.06	−9650.83	−9245.67
Num. obs.	40133	20183	40133	23826	40133	15756
Num. groups: week	13	13	13	13	13	13
Num. groups: token	4283	1999	4283	2326	4283	1558
Var: week (Intercept)	0.15	0.12	0.22	0.14	0.21	0.24
Var: token (Intercept)	0.44	0.10	0.70	0.09	0.68	0.02

$^{*}p < 0.05$, All estimates as log odds, own calculations.

Table A11: Replication of Table A3 including all hygiene measures: Logit and count models for infections in children, parents and staff, wave 5

	(1): Child, 5 occurrence	(2): Child, 5. number	(3): Parent, 5. occurrence	(4): Parent, 5. number	(5): Staff, 5 occurrence	(6): Staff, 5 number
Intercept	−2.58*	−1.16*	−1.98*	−1.01*	−1.98*	−1.35*
	[−3.16; −2.01]	[−1.61; −0.70]	[−2.74; −1.22]	[−1.50; −0.52]	[−2.53; −1.43]	[−1.76; −0.93]
SES: 11 to 30% of low SES children (ref.: below 10%)	0.11*	0.05*	−0.01	−0.05	0.11*	0.08*
	[0.05; 0.18]	[0.00; 0.10]	[−0.10; 0.08]	[−0.11; 0.01]	[0.05; 0.18]	[0.03; 0.13]
SES: 31 to 60% of low SES children	0.10*	−0.01	−0.13*	−0.09*	0.22*	0.16*
	[0.00; 0.19]	[−0.08; 0.06]	[−0.26; −0.00]	[−0.17; −0.01]	[0.13; 0.31]	[0.10; 0.23]
SES: above 60% of low SES children	0.03	−0.13*	−0.17*	−0.07	0.35*	0.31*
	[−0.09; 0.15]	[−0.21; −0.04]	[−0.34; −0.01]	[−0.17; 0.03]	[0.23; 0.46]	[0.23; 0.39]
Regular ventilation of rooms (between)	0.43	0.35	0.52	0.20	−0.01	0.13
	[−0.10; 0.96]	[−0.07; 0.77]	[−0.19; 1.23]	[−0.25; 0.66]	[−0.51; 0.49]	[−0.25; 0.50]
Regular ventilation of rooms (within)	0.09	0.15	0.09	0.15	0.08	0.07
	[−0.24; 0.43]	[−0.11; 0.41]	[−0.26; 0.44]	[−0.10; 0.40]	[−0.26; 0.42]	[−0.20; 0.34]
Fixed staff assignment to groups (between)	−0.01	−0.03	−0.16*	−0.03	−0.02	0.01
	[−0.11; 0.08]	[−0.10; 0.04]	[−0.29; −0.02]	[−0.11; 0.05]	[−0.11; 0.07]	[−0.06; 0.07]
Fixed staff assignment to groups (within)	0.10	0.09*	0.05	0.04	−0.11	−0.07
	[−0.01; 0.22]	[0.01; 0.17]	[−0.06; 0.17]	[−0.04; 0.12]	[−0.22; 0.01]	[−0.15; 0.02]
Group separation indoors (between)	0.18*	0.03	0.23*	0.02	0.08	−0.06
	[0.07; 0.28]	[−0.05; 0.11]	[0.09; 0.38]	[−0.07; 0.11]	[−0.03; 0.18]	[−0.13; 0.02]
Group separation indoors (within)	0.09	0.07	0.02	0.05	0.04	−0.01
	[−0.02; 0.21]	[−0.01; 0.16]	[−0.10; 0.14]	[−0.04; 0.13]	[−0.08; 0.16]	[−0.10; 0.08]
Group separation outdoors (between)	−0.13*	0.01	−0.14*	−0.00	−0.09	−0.02
	[−0.22; −0.03]	[−0.05; 0.08]	[−0.27; −0.01]	[−0.08; 0.08]	[−0.18; 0.00]	[−0.09; 0.04]
Group separation outdoors (within)	−0.10	−0.10*	−0.01	−0.02	0.02	0.06
	[−0.24; 0.03]	[−0.19; −0.00]	[−0.14; 0.13]	[−0.12; 0.07]	[−0.12; 0.15]	[−0.04; 0.17]
Regular surface disinfection (between)	0.05	0.01	0.12	0.09*	−0.03	0.03
	[−0.04; 0.15]	[−0.06; 0.08]	[−0.01; 0.26]	[0.01; 0.17]	[−0.13; 0.06]	[−0.04; 0.10]
Regular surface disinfection (within)	−0.08	−0.01	0.00	−0.04	0.01	−0.04
	[−0.23; 0.07]	[−0.11; 0.10]	[−0.15; 0.15]	[−0.15; 0.06]	[−0.14; 0.15]	[−0.15; 0.07]
Temperature measurement for staff (between)	0.16	0.15	0.35	0.00	−0.07	−0.05
	[−0.13; 0.44]	[−0.06; 0.36]	[−0.04; 0.74]	[−0.24; 0.24]	[−0.35; 0.21]	[−0.25; 0.16]
Temperature measurement for staff (within)	0.20	0.23	0.28	0.17	0.29	0.22
	[−0.18; 0.58]	[−0.04; 0.49]	[−0.10; 0.65]	[−0.09; 0.42]	[−0.08; 0.67]	[−0.07; 0.50]
Temperature measurement for children (between)	0.02	−0.04	−0.03	0.06	0.04	−0.06
	[−0.17; 0.22]	[−0.18; 0.10]	[−0.29; 0.24]	[−0.10; 0.23]	[−0.15; 0.23]	[−0.20; 0.07]
Temperature measurement for children (within)	−0.04	−0.07	−0.20	−0.15	−0.35*	−0.13
	[−0.36; 0.28]	[−0.30; 0.17]	[−0.53; 0.13]	[−0.38; 0.08]	[−0.68; −0.02]	[−0.38; 0.13]
Staff face mask with staff/parents (between)	0.02	−0.03	0.09	0.06	0.19*	0.11*
	[−0.07; 0.12]	[−0.10; 0.04]	[−0.05; 0.22]	[−0.02; 0.14]	[0.09; 0.28]	[0.04; 0.18]
Staff face mask with staff/parents (within)	0.09	−0.03	0.08	0.06	−0.06	−0.07
	[−0.04; 0.22]	[−0.12; 0.07]	[−0.05; 0.22]	[−0.04; 0.15]	[−0.19; 0.07]	[−0.17; 0.03]
Staff face mask with children (between)	0.09*	0.08*	0.08	0.08*	−0.06	−0.12*
	[0.02; 0.16]	[0.02; 0.13]	[−0.02; 0.18]	[0.02; 0.14]	[−0.13; 0.01]	[−0.17; −0.07]
Staff face mask with children (within)	−0.08	−0.15*	−0.05	−0.11*	−0.10	−0.12*
	[−0.18; 0.02]	[−0.22; −0.07]	[−0.15; 0.06]	[−0.18; −0.03]	[−0.21; 0.00]	[−0.20; −0.04]
Test staff for COVID-19 (between)	0.12	0.09	0.10	0.03	0.06	−0.06
	[−0.03; 0.27]	[−0.02; 0.20]	[−0.11; 0.31]	[−0.10; 0.16]	[−0.09; 0.20]	[−0.17; 0.04]
Test staff for COVID-19 (within)	0.04	0.08	0.03	0.02	0.14	0.13*
	[−0.11; 0.18]	[−0.03; 0.19]	[−0.12; 0.18]	[−0.09; 0.12]	[−0.01; 0.29]	[0.01; 0.24]
Test children for COVID-19 (between)	0.13*	0.04	0.04	0.02	0.07	0.01
	[0.03; 0.22]	[−0.03; 0.11]	[−0.09; 0.17]	[−0.05; 0.10]	[−0.02; 0.16]	[−0.05; 0.08]
Test children for COVID-19 (within)	0.03	0.07	0.02	0.03	0.03	0.05
	[−0.08; 0.13]	[−0.01; 0.15]	[−0.09; 0.13]	[−0.04; 0.11]	[−0.08; 0.14]	[−0.04; 0.13]
Vaccination rate (between)	0.14*	−0.03	0.19*	0.05	−0.05	−0.03
	[0.02; 0.26]	[−0.12; 0.05]	[0.02; 0.36]	[−0.06; 0.15]	[−0.17; 0.07]	[−0.12; 0.05]
Vaccination rate (within)	−0.09	0.05	0.03	0.11	−0.20	−0.06
	[−0.34; 0.17]	[−0.13; 0.24]	[−0.23; 0.28]	[−0.08; 0.30]	[−0.47; 0.06]	[−0.26; 0.14]
Nr. of Infections: Staff (between)	0.62*	0.59*	0.13*	0.15*		
	[0.55; 0.68]	[0.55; 0.63]	[0.04; 0.22]	[0.09; 0.20]		
Nr. of Infections: Staff (within)	0.17*	0.14*	0.01	0.04*		
	[0.14; 0.19]	[0.12; 0.15]	[−0.01; 0.04]	[0.02; 0.06]		
Nr. of Infections: Parents (between)	0.37*	0.18*			0.13*	0.04*
	[0.33; 0.40]	[0.16; 0.20]			[0.10; 0.16]	[0.02; 0.06]
Nr. of Infections: Parents (within)	0.11*	0.05*			0.03*	0.02*
	[0.09; 0.13]	[0.04; 0.06]			[0.01; 0.04]	[0.01; 0.03]
7 day Incidence (within)	0.00*	0.00*	0.00*	0.00*	0.00*	0.00*
	[0.00; 0.00]	[0.00; 0.00]	[0.00; 0.00]	[0.00; 0.00]	[0.00; 0.00]	[0.00; 0.00]
7 day Incidence (between)	0.00*	−0.00	0.00	−0.00	0.00*	0.00
	[0.00; 0.00]	[−0.00; 0.00]	[−0.00; 0.00]	[−0.00; 0.00]	[0.00; 0.00]	[−0.00; 0.00]
Increase 7 day Incidence (t to t+x, within)	0.00*	0.00*	0.00*	0.00*	0.00*	0.00*
	[0.00; 0.00]	[0.00; 0.00]	[0.00; 0.00]	[0.00; 0.00]	[0.00; 0.00]	[0.00; 0.00]
Increase 7 day Incidence (t to t+x, between)	0.00*	0.00*	0.00	0.00*	0.00*	0.00*
	[0.00; 0.00]	[0.00; 0.00]	[−0.00; 0.00]	[0.00; 0.00]	[0.00; 0.00]	[0.00; 0.00]
Nr. of Infections: Children (between)			0.15*	0.20*	0.24*	0.23*
			[0.11; 0.19]	[0.18; 0.23]	[0.21; 0.27]	[0.22; 0.25]
Nr. of Infections: Children (within)			0.01	0.02*	0.06*	0.04*
			[−0.00; 0.02]	[0.01; 0.03]	[0.05; 0.07]	[0.03; 0.05]
AIC	38747.64	94884.82	39395.89	82190.37	38047.60	68293.44
Log Likelihood	−19329.82	−47397.41	−19653.95	−41050.19	−18979.80	−34101.72
Num. obs.	31030	30037	31030	28498	31030	29430
Num. groups: week	11	11	11	11	11	11
Num. groups: token	4073	3821	4073	3566	4073	3683
Var: week (Intercept)	0.03	0.04	0.03	0.02	0.05	0.04
Var: token (Intercept)	0.24	0.12	0.96	0.24	0.19	0.04

$^{*}p < 0.05$, All estimates as log odds, own calculations.
